# Physical Fitness Dynamics Shape Immune Remodeling in Healthy Aging: A 3‐Year Longitudinal Study

**DOI:** 10.1111/acel.70440

**Published:** 2026-03-06

**Authors:** Christopher Weyh, Vincent Größer, Luciele Guerra Minuzzi, Torsten Frech, Kristina Gebhardt, Svenja Nolte, Theresa Dombrowski, Manuela Andrea Hoffmann, Natascha Sommer, Robert Ringseis, Klaus Eder, Samuel Sossalla, Pascal Bauer, Karsten Krüger

**Affiliations:** ^1^ Department of Exercise Physiology and Sports Therapy, Institute of Sports Science Justus‐Liebig‐University Giessen Germany; ^2^ Department of Cardiology and Angiology Justus‐Liebig‐University Giessen Giessen Germany; ^3^ CIPER, Faculty of Sport Sciences and Physical Education University of Coimbra Coimbra Portugal; ^4^ Institute for Preventive Medicine of the German Armed Forces Andernach Germany; ^5^ Department of Nuclear Medicine University Medical Center of the Johannes Gutenberg‐University Mainz Germany; ^6^ Department of Internal Medicine Universities of Giessen and Marburg Lung Center (UGMLC), Member of the German Center for Lung Research (DZL), Excellence Cluster Cardio‐Pulmonary Institute (CPI), Justus‐Liebig University Giessen Germany; ^7^ Institute of Animal Nutrition and Nutrition Physiology Justus Liebig University Giessen Giessen Germany; ^8^ Center for Sustainable Food Systems Justus Liebig University Giessen Giessen Germany; ^9^ Department of Cardiology Kerkhoff Clinic GmbH Bad Nauheim Germany

**Keywords:** healthy aging, immune aging, physical fitness, T cells

## Abstract

Aging is accompanied by functional decline and immune remodeling, yet the dynamics and early modifiability of these processes remain incompletely understood. Research suggests that lifestyle factors, particularly physical activity and fitness, influence immune aging. This study investigated longitudinal changes in physical performance and immune parameters in a well‐characterized cohort of clinically healthy elderly. In this study, 49 clinically healthy elderly underwent repeated assessments of cardiorespiratory fitness, muscular strength, body composition, immune cell phenotypes, and serum cytokines at baseline, 1‐year, and 3‐year follow‐up. We observed a shift toward an aged T cell profile, characterized by reductions in naïve and regulatory T cells (Tregs), alongside increases in differentiated and senescence‐associated subsets. Treg subsets followed divergent trajectories, with resting Tregs (rTregs) declining and memory‐like Tregs (mTregs) increasing. Serum levels of classical pro‐inflammatory cytokines remained largely stable over the study period. Despite stable self‐reported physical activity, participants showed declines in cardiorespiratory fitness and strength. Immune remodeling was primarily associated with declines in physical fitness, alongside an increase in highly differentiated CD4^+^ and senescent CD8^+^ T cell subsets, lower rTregs, higher mTregs, and increased CD4^−^CD8^−^ lymphocyte frequencies, while habitual physical activity was independently related to effector T cell dynamics. Together, these findings indicate that subtle functional decline in clinically healthy older adults is paralleled by immune changes characteristic of early immunosenescence, occurring largely in the absence of overt systemic inflammation. These results highlight physical fitness as a potentially modifiable determinant of immune trajectories and immune resilience in healthy aging.

## Graphical abstract

In a 3‐year longitudinal study of healthy older adults, intraindividual declines in physical fitness and strength were linked to dynamic immune remodeling, including shifts toward senescent and regulatory T cell phenotypes, despite stable activity levels. These findings identify physical fitness as a key, potentially modifiable driver of early and subclinical immune aging.

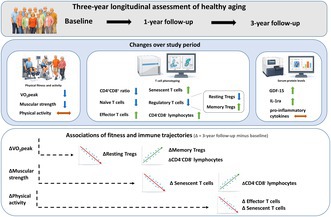

## 1 Introduction

Aging is accompanied by a progressive decline in physiological function, including profound alterations in immune regulation (Yousefzadeh et al. 2021; Taffet 2020). A key hallmark of this process is immunosenescence, defined by shifts in T cell phenotypes, diminished adaptive immunity, and impaired responses to novel antigens. Although the overall number of T cells remains relatively stable throughout adulthood, aging is characterized by a pronounced redistribution of T cell subsets (Wikby et al. 2008). The proportion of CD8^+^ T cells increases, while CD4^+^ T cells decline, resulting in a reduced CD4^+^/CD8^+^ ratio, a central feature of the so‐called immune risk profile. Furthermore, the number of CD4^+^ and CD8^+^ naïve T cells (expressing surface receptors such as CD45RA^+^) decrease, and more differentiated T cells accumulate due to thymic involution and chronic antigenic stimulation (Pawelec et al. 2020). Senescent T cells secrete pro‐inflammatory cytokines (Davalos et al. 2010), thereby contributing “inflammaging” a state of chronic, low‐grade systemic inflammation (Fülöp et al. 2016), characterized by elevated levels of interleukin‐6 (IL‐6), tumor necrosis factor‐α (TNF‐α), and interleukin‐1β (IL‐1β) (Minciullo et al. 2016). Another important regulatory component in this context is the population of regulatory T cells (Tregs), which help maintain immune homeostasis by suppressing excessive immune activation. Tregs, largely generated in the thymus, exert their effects through cell‐contact dependent mechanisms and the secretion of anti‐inflammatory cytokines such as IL‐10 and Transforming Growth Factor‐beta (TGF‐β) (Dikiy and Rudensky 2023). Reduced number or function of Tregs and their subsets has been implicated in chronic inflammation, autoimmune diseases, and the progression of atherosclerosis (Sumida et al. 2024; Böttrich et al. 2023). Clinically, all these immune alterations are associated with higher incidence and poorer outcomes of infections, reduced vaccine efficacy, and increased prevalence of chronic noncommunicable diseases such as atherosclerosis, type 2 diabetes, and cancer in older adults (Liu et al. 2023). Together, these changes constitute the basis of immune aging—a key mechanism linking advancing age to increased vulnerability to severe infections and noncommunicable diseases. However, this is not a deterministic outcome. An increasing body of research supports the idea that lifestyle factors—most notably physical activity—can influence the process of immune aging (Duggal et al. 2019). Exercise has emerged as a potent modulator of immune function. Regular physical activity is associated with lower systemic inflammation, improved immune surveillance, and the preservation of immunological diversity (Weyh et al. 2020). Studies have shown that physical activity can attenuate the decline of naïve T cells, reduce the accumulation of senescent phenotypes, and is associated with frequency of Tregs and their subsets (Böttrich et al. 2023; Despeghel et al. 2021; Proschinger et al. 2021). Moreover, physical activity exerts robust anti‐inflammatory effects, including reductions in circulating pro‐inflammatory cytokines such as IL‐6 and TNF‐α, which are central mediators of inflammaging (Bautmans et al. 2021). These effects may translate into better immune resilience and protection against inflammation‐related morbidity. Previous work in the same cohort identified distinct immune signatures associated with subclinical vascular alterations, highlighting the sensitivity of immune parameters as early indicators of biological aging (Böttrich et al. 2023; Weyh et al. 2025). However, much of the existing evidence is derived from cross‐sectional studies or short‐term interventions and therefore provides limited insight into how intraindividual changes in physical fitness over time relate to immune remodeling during aging. Critically, it remains unclear whether age‐related immune changes are primarily associated with activity behavior or with objective declines in physiological capacity, such as cardiorespiratory fitness and muscular strength. Moreover, longitudinal data examining whether changes in physical fitness are accompanied by coordinated adaptations within regulatory and senescence‐associated immune compartments are scarce. To our knowledge, no previous studies have longitudinally examined intraindividual changes in physical fitness and detailed immune phenotypes over a comparable multiyear period in clinically healthy older adults. Therefore, this study aimed to extend current knowledge by exploring longitudinal intraindividual associations between changes in physical performance and immune cell compositions over a 3‐year period in a well‐characterized cohort of healthy older adults. By integrating repeated measures of cardiorespiratory fitness, muscular strength, body composition, and detailed immune phenotyping in a longitudinal design, this study determine whether physical fitness trajectories, rather than static fitness levels, are associated to distinct patterns of immune aging remodeling. Focusing on early immune changes in the absence of overt disease, this study seeks to advance our understanding of physical fitness and activity as a dynamic determinant of immune aging and resilience.

## 2 Methods

### 2.1 Study Design and Participants

This longitudinal observational study was conducted between August 2020 and December 2024 as part of the Giessen Immune Aging Study. Participants were recruited via institutional e‐mail distribution lists of Justus Liebig University Giessen, online announcements, local television features, and postings in healthcare facilities. Interested individuals underwent a standardized telephone‐based prescreening during which predefined inclusion and exclusion criteria were assessed. No additional selection procedures were applied. The cohort consisted of 49 healthy older adults (male: *n* = 30; female: *n* = 19) with a mean baseline age of 63.8 ± 3.8 years. All participants were free from acute or chronic diseases, including infectious, cardiovascular, metabolic, or inflammatory conditions, and reported no regular medication use throughout the study period. Detailed inclusion and exclusion criteria have been published previously (Böttrich et al. 2023). All participants completed a standardized 3‐year follow‐up. The present analysis builds upon the same cohort that was previously characterized in cross‐sectional investigations (Böttrich et al. 2023; Weyh et al. 2025). The current analysis focuses on longitudinal trajectories of immune aging and their relationship to physiological adaptations across the entire cohort, independent of the vascular findings reported in previous cross‐sectional studies, in order to capture systemic aspects of healthy aging. Detailed anthropometric and clinical baseline characteristics are summarized in Table 1. Participants underwent follow‐up assessments at 1‐ and 3‐year after baseline. At each time point, anthropometric measurements, physical performance tests, and detailed immunological analyses including cytokine profiling and flow‐cytometric characterization of T cell subsets were performed. The study protocol was approved by the Ethics Committee of the Justus Liebig University Giessen (Ref. No. 100/20), and all procedures were conducted in accordance with the Declaration of Helsinki. Written informed consent was obtained from all participants prior to enrollment. A clinical trial registration number is not applicable due to the non‐interventional design of this observational study. Given the exploratory and observational nature of this longitudinal study, no formal a priori sample size or power calculation was performed prior to recruitment.

**TABLE 1:** Longitudinal changes in anthropometric, physical performance, physical activity, T cell subset distributions and regulatory/effector balance, circulating inflammatory and regulatory cytokines parameters in healthy older adults over a 3‐year follow‐up.

	Baseline	1‐year follow‐up	3‐year follow up	*p*
BMI (kg/m^2^)	25.0 ± 3.2	24.8 ± 3.2	24.8 ± 3.2	0.228
Body fat (%)	27.7 ± 7.0	26.0 ± 7.1	28.4 ± 6.7	**< 0.001** ^a^, ^b^
Visceral fat (%)	30.7 ± 6.2	28.3 ± 6.6	31.5 ± 6.1	**< 0.001** ^a^, ^b^
VO_2peak_ (mL/kg/min)	30.6 ± 7.1	29.86 ± 6.7	28.8 ± 7.0	**0.006** ^c^
Grip strength (kg)	44.4 ± 10.1	43.0 ± 10.5	38.8 ± 10.5	**< 0.001** ^a^, ^b^, ^c^
Leg extension (Nm)	296.3 ± 109.8	309.8 ± 100.8	294.3 ± 96.6	0.202
Leg flexion (Nm)	126.7 ± 49.0	111.1 ± 34.4	110.5 ± 34.9	**0.002** ^a^, ^c^
Total MET's (min/week)	5567.0 ± 3152.4	5776.5 ± 4967.6	5415.8 ± 3739.5	0.897
CD4^+^/CD8^+^ Ratio	2.0 ± 1.4	1.4 ± 0.9	1.4 ± 1.1	**0.002** ^a^, ^c^
CD4^+^ Naïve (%)	36.4 ± 16.9	31.0 ± 15.5	27.7 ± 15.6	**< 0.001** ^a^, ^c^
CD4^+^ CM (%)	25.6 ± 11.3	25.7 ± 9.1	29.0 ± 11.8	**0.017** ^b^, ^c^
CD4^+^ EM (%)	32.8 ± 16.3	38.3 ± 15.3	38.7 ± 16.0	0.066
CD4^+^ EMRA (%)	5.3 ± 7.7	4.36 ± 6.4	4.15 ± 6.5	0.390
CD4^+^CD57^+^ (%)	8.9 ± 13.0	9.9 ± 14.2	9.2 ± 14.0	0.074
CD8^+^ Naïve (%)	14.6 ± 15.9	6.5 ± 5.1	6.1 ± 5.0	**< 0.001** ^a^, ^c^
CD8^+^ CM (%)	5.3 ± 6.5	3.7 ± 2.6	2.9 ± 2.7	**0.009** ^b^, ^c^
CD8^+^ EM (%)	32.3 ± 14.9	33.8 ± 13.0	36.9 ± 14.2	0.131
CD8^+^ EMRA (%)	44.2 ± 18.6	53.7 ± 14.5	55.4 ± 14.0	**< 0.001** ^a^, ^c^
CD8^+^ CD57^+^ (%)	39.9 ± 14.6	44.2 ± 14.8	46.2 ± 15.3	**0.004** ^c^
Teff (%)	75.8 ± 7.0	78.3 ± 6.8	82.5 ± 10.3	**< 0.001** ^a^, ^b^, ^c^
Treg (%)	6.0 ± 1.6	4.1 ± 1.4	2.9 ± 1.5	**< 0.001** ^a^, ^b^, ^c^
Treg/Teff ratio	0.08 ± 0.02	0.05 ± 0.02	0.03 ± 0.02	**< 0.001** ^a^, ^b^, ^c^
rTreg (%)	32.7 ± 10.7	22.8 ± 9.4	10.1 ± 7.8	**< 0.001** ^a^, ^b^, ^c^
mTreg (%)	31.4 ± 13.1	45.8 ± 9.00	56.0 ± 20.1	**< 0.001** ^a^, ^b^, ^c^
CD4^−^CD8^−^ lymphocytes (%)	23.4 ± 6.8	25.7 ± 9.2	27.2 ± 10.0	**0.026** ^c^
CCL‐2 (pg/mL)	496.6 ± 156.2	503.2 ± 167.3	487.80 ± 155.8	0.480
CXCL‐9 (pg/mL)	863.7 ± 1328.1	864.4 ± 1389.1	846.3 ± 1344.6	0.785
CXCL‐10 (pg/mL)	12.2 ± 7.3	13.1 ± 8.6	12.1 ± 8.2	0.801
GDF‐15 (pg/mL)	573.1 ± 309.7	614.3 ± 372.0	626.1 ± 345.3	**0.004** ^a^, ^c^
IL‐1ra (pg/mL)	726.6 ± 1146.3	793.0 ± 1193.3	822.7 ± 1242.8	**0.023** ^c^
IL‐6 (pg/mL)	19.2 ± 38.4	20.04 ± 41.1	25.0 ± 61.0	0.326
IL‐10 (pg/mL)	1.8 ± 1.4	2.1 ± 1.4	1.9 ± 1.5	0.097
IL‐18 (pg/mL)	502.3 ± 269.6	499.6 ± 506.2	506.2 ± 298.7	0.852
TNF‐α (pg/mL)	6.4 ± 5.4	6.6 ± 5.6	6.6 ± 5.9	0.711
VEGF (pg/mL)	520.9 ± 585.6	562.0 ± 612.7	499.5 ± 403.9	0.085

*Note:* Bold values indicate statistical significance (*p* < 0.05).

Abbreviations: BMI, body mass index; CCL, C‐C motif chemokine ligand; CD, cluster of differentiation; CM, central memory T cells; CXCL, C‐X‐C motif chemokine ligand; DN, double‐negative T cells; EM, effector memory T cells; EMRA, effector memory T cells re‐expressing CD45RA; GDF, growth differentiation factor; IL, interleukin; IL‐1ra, interleukin‐1 receptor antagonist; MET, metabolic equivalent of task; mTreg, memory Treg cells; Nm, Newton meter; rTreg, naïve Treg cells; Teff, T effector cells; TNF‐α, tumor necrosis factor‐alpha; Treg, regulatory T cells; VEGF, vascular endothelial growth factor; VO_2peak_, peak oxygen uptake.

^a^ Indicates post hoc differences between baseline and 1‐year follow‐up.

^b^ Post hoc differences between 1‐year follow‐up and 3‐year follow‐up.

^c^ Post hoc differences between baseline and 3‐year follow‐up.

### 2.2 Baseline Characteristics

At all three study visits, venous fasting blood samples were collected from each participant between 08:00 and 10:00 AM under standardized conditions. Participants were instructed to refrain from exercising the day before the appointment. Samples were processed for a range of biochemical and immunological analyses, including the isolation of peripheral blood mononuclear cells (PBMCs) and the collection of serum for protein signature profiling. Fasting serum concentrations of glucose, insulin, total cholesterol, low‐density lipoprotein (LDL), high‐density lipoprotein (HDL), triglycerides, and cortisol were determined using standard clinical laboratory procedures at SYNLAB Medical Care Center (Bad Nauheim, Germany). Body composition was assessed consistently across all time points using bioelectrical impedance analysis (BIA) with the BIACORPUS RX 4004 M device and BodyComposition—Professional Version 9.0.20413 software (MEDI CAL HealthCare GmbH). The analysis included body fat percentage (%) and visceral fat (%).

### 2.3 Isolation of Peripheral Blood Mononuclear Cells

To isolate peripheral blood mononuclear cells (PBMCs), fresh peripheral blood was diluted 1:1 with PBS (phosphate‐buffered saline) and layered onto EasySept (STEMCELL Technologies, Vancouver, Canada) using SepMate (STEMCELL Technologies, Vancouver, Canada) 50 mL tubes. After centrifugation at 1200 × g for 10 min, the upper layer was carefully poured off. The isolated cells were then washed and centrifuged for 8 min at 300 × g. Subsequently, the PBMCs were frozen down by resuspending them in freezing medium called Bambanker (Nippon Genetics Europe GmbH, Düren, Germany). The frozen cells were stored at −80°C for future analysis.

### 2.4 T Cell Phenotyping by Flow Cytometry

To analyze T cells and their subsets, frozen PBMCs were thawed at 37°C, washed twice in RPMI 1640 (Gibco, UK) containing 10% FBS (Gibco, UK), and then pelleted. The amount of cells was divided in two samples according to two different panels used. For Panel 1 (Immune aging panel) and Panel 2 (Treg panel) fluorescence staining was performed directly after resting time. For panel 1 cells were resuspended in PBS at a concentration of 1 × 10^6^ cells/mL and stained with fluorescence Zombie Aqua fixable viability kit, CD4 (FITC), CD8 (AF700), CD197/CCR7 (BV421), CD45RA (APC), and CD57 (PE). For panel 2 cells were also resuspended in PBS at a concentration of 1 × 10^6^ cells/mL and stained with fluorescence Zombie Aqua fixable viability kit, CD3 (FITC), CD4 (AF700), CD25 (APC), CD45RO (PerCP/Cyanine5.5), CD197/CCR7 (BV421), and CD127 (PE) all from BioLegend (BioLegend Inc., San Diego, CA). For both panels the gating strategy was first selected lymphocytes based on forward scatter/side scatter (FSC/SSC) in a dot plot, with a minimum of 500,000 events per tube. Then singlets were identified within the lymphocyte gate, followed by the identification of live cells. For the Panel 1 CD4^+^ and CD8^+^ T cells were gated after determination of live cells (Zombie NIR fixable viability kit) for CCR7‐CD45RA to analyze the abundance of naïve T cells (CD45RA^+^CCR7^+^), effector memory (EM) T cells (CD45RA^−^CCR7^−^), central memory (CM) T‐cells (CD45RA^−^CCR7^+^), and effector memory cells re‐expressing CD45RA T cells (TEMRA) (CD45RA^+^CCR7^−^). Additionally, double‐negative CD4^−^CD8^−^ lymphocytes were identified within the lymphocyte gate lacking both CD4 and CD8 expression after exclusion of nonviable cells. Terminal differentiated T cells were gated using CD57^+^. For the gating strategy to identify regulatory T cells the frequency of CD3^+^CD4^+^ cells was evaluated after determining live cells (Zombie NIR fixable viability kit). T effector cells (Teff) were gated as CD4^+^CD25^+^CD127_high_, while Tregs were defined as CD4^+^CD25^+^CD127_low_. Tregs subsets were further gated as naïve (rTregs; CD45RO^−^CCR7^+^) and memory (mTregs; CD45RO^+^CCR7^−^) Tregs. For detailed gating strategy, see Figures S1 and S2. The ratio of Tregs to Teff was calculated by dividing the total number of Tregs by the total number of Teff cells. Flow cytometry analysis was performed using a CytoFLEX S (Beckman Coulter) and Kaluza analysis software 2.3 (Beckman Coulter), with compensation applied for spectral overlap when using multiple colors.

### 2.5 Serum Protein Signatures and Cytomegalovirus (CMV) Serostatus

Serum was stored at −80°C until analysis. Proteins were determined using a human Magnetic Luminex Assay (Bio‐Techne, Abingdon, Oxon, UK) and a Luminex 200 system (Luminex Corp, Austin, TX, USA) according to the manufacturer's instructions. In total, 10 different proteins involved in inflammation were determined for protein signature analysis: C‐C motif chemokine ligand 2 (CCL2), C‐X‐C chemokine ligand (CXCL)‐9, CXCL‐10, growth differentiation factor (GDF)‐15, interleukin (IL)‐1 receptor antagonist (ra), IL‐6, IL‐10, IL‐18, tumor necrosis factor (TNF)‐alpha (α), and the vascular endothelial growth factor (VEGF). Serum anti‐CMV immunoglobulin G (IgG) antibodies were detected at baseline and 3‐year follow‐up using a semiquantitative sandwich enzyme‐linked immunosorbent assay (ELISA‐Viditest anti‐CMV IgG, VIDIA, Czech Republic). The procedure followed the manufacturer's instructions. End‐point optical density was measured by ELISA reader SPECTROstar Nano (BMG Labtech, Germany).

### 2.6 Measurements of Cardiorespiratory Fitness, Muscle Strength and Physical Activity

Cardiopulmonary exercise testing (CPET) was performed on an electronically braked cycle ergometer (Excalibur Sport, Lode) using two standardized ramp protocols. Following a 3‐min warm‐up period without resistance, the specific protocol was selected according to the participant's training and fitness status, aiming to reach maximal load within ~15 min. Prior to testing, participants were assessed for their sports history. Participants without systematic endurance training or competition experience completed a two‐step ramp protocol: an initial 3‐min unloaded phase, followed by cycling at 50 watts (W) with increments of 25 W every 3 min until 100 W, after which the workload was increased by 25 W every 2 min. Trained subjects started at 50 W after the 3‐min warm‐up period without load and increased by 50 W every 3 min. The test was performed until complete exhaustion. The following criteria were used to verify exhaustion: request of the participant due to extreme tiredness and/or perception of the intense dyspnea; reached the maximum heart rate (HR) predicted by age (HR max) was ≥ 85%; peak respiratory exchange ratio RER > 1.1; VO_2_ plateau was reached even with increasing workload. Ventilatory and metabolic parameters were collected by respiration using Metalyzer 3‐B (Cortex, Germany) and were analyzed. The average of the last test 30 s was used to determine the VO_2peak_. Maximum voluntary grip strength of the dominant hand was measured using a hand grip dynamometer (Baseline Hydraluic Hand Dynamometer LiTE, Fabrication Enterprises Inc., US). Participants were instructed to keep their arms by their side during the assessment. Verbal encouragement was provided to motivate the participants to exert their maximum effort. Three maximal contractions were performed, and the highest recorded grip strength value was used for subsequent analysis. Maximum isometric leg strength was measured by peak isometric torque (Newton meter [Nm]) during knee flexion and extension by the m3‐Diagnos analysis station (Schnell). Knee extension was measured at a knee joint angle of 60°, whereas knee flexion was measured at a knee joint angle of 90°. Three trials were completed for each position, with contractions lasting 5 s, separated by 30 s rest intervals. Participants were encouraged verbally to elicit their maximal effort. Peak torque values (Nm) were recorded, and the highest of the repetitions was used for statistical analysis. Physical activity was determined by using a self‐administered form of the international physical activity questionnaire (IPAQ) long form. The questionnaire assesses physical activity PA carried out in the past 7 days. Participants were asked to report all activities performed for at least 10 min during that period. Detailed information was collected in a second step about according to days per week and hours per day. The energy expenditure indicator metabolic equivalent of task (MET) per week was calculated by multiplying the number of minutes per week of each activity‐based metabolic cost. METs are commonly used to express the intensity of physical activities and are also used for the analysis of our data.

### 2.7 Data Analysis

All statistical analyses were conducted using IBM SPSS Statistics (Version 29.0, IBM Corp., Armonk, NY) and Prism (Version 10, GraphPad Software, San Diego, CA) for graphical visualization. First, the normality of the distribution of each variable was assessed using the Shapiro–Wilk test. Depending on the distribution, parametric or nonparametric procedures were applied. To examine changes over time across the three measurement points (baseline, 1‐year follow‐up, 3‐year follow‐up), repeated‐measures ANOVA or the Friedman test (for non‐normally distributed variables) was applied. To evaluate group effects and potential interactions with time, a two‐way repeated‐measures ANOVA was performed with sex and CMV serostatus as between‐subject factors and time as the within‐subject factor. Main effects and interaction terms were of particular interest. Post hoc comparisons were adjusted using the Bonferroni–Holm correction. Subsequently, changes (Δ‐values) between baseline and 3‐year follow‐up were calculated to assess interindividual variation in longitudinal changes. Bivariate associations between variables were examined using Pearson's correlation coefficient (r) for normally distributed variables or Spearman's rank correlation coefficient (ρ) otherwise. To further evaluate the most prominent associations identified in the correlation analyses, multiple linear regression models were performed. A stepwise hierarchical approach was used, comprising five steps: starting with a univariate regression model, covariates were added stepwise; baseline age (Model 2), sex (Model 3), CMV serostatus (Model 4), and visceral fat change as an anthropometric factor (Model 5). Standardized regression coefficients (β) and 95% confidence intervals (CI) are reported. Descriptive data are presented as means and standard deviations (M ± SD), unless otherwise indicated. Given the exploratory nature of the analyses, no correction for multiple testing in the outcomes was applied.

## 3 Results

Table 1 presents longitudinal changes in anthropometric and physical performance parameters, cellular immune parameters, and cytokine levels across baseline, one‐year, and three‐year follow‐up assessments. Body fat percentage (*F* (1.76, 84.41) = 15.61, *p* < 0.001) and visceral fat percentage (*F* (2.00, 96.00) = 18.78, *p* < 0.001) decreased from baseline to 1‐year follow‐up and increased again by 3‐year follow‐up. Cardiorespiratory fitness, assessed by VO_2peak_, declined over the observation period (*F* (1.75, 83.77) = 5.75, *p* = 0.006), accompanied by reductions in grip strength (*F* (2.00, 96.00) = 55.11, *p* < 0.001) and leg flexion strength (*F* (1.69, 79.17) = 6.95, *p* = 0.003).

The CD4^+^/CD8^+^ ratio declined from baseline to one‐year follow‐up and remained reduced at 3 years (*F* (2.00, 96.00) = 6.67, *p* = 0.002). Similarly, naïve CD4^+^ T cells declined progressively over time (*F* (2.00, 96.00) = 8.32, *p* < 0.001), whereas CD4^+^ CM T cells increased between 1‐ and 3‐year follow‐up (*F* (2.00, 96.00) = 4.26, *p* = 0.017). Within the CD8^+^ subset, naïve CD8^+^ T cells decreased from baseline to 1 year and remained low at 3 years (*F* (1.19, 57.25) = 16.51, *p* < 0.001), while CD8^+^ EMRA T cells increased (*F* (1.43, 68.66) = 9.89, *p* < 0.001). CD8^+^ CM T cells decreased (*F* (1.31, 62.69) = 5.81, *p* = 0.009), and CD8^+^CD57^+^ cells increased from 1‐ to 3‐year follow‐up (*F* (2.00, 96.00) = 5.80, *p* = 0.004). Regarding functional T cell subsets, Teff increased across all time points (*F* (1.54, 73.70) = 17.50, *p* < 0.001), while Treg declined continuously (*F* (1.69, 81.10) = 83.40, *p* < 0.001), resulting in a marked reduction in the Treg/Teff ratio (*F* (1.72, 70.61) = 97.90, *p* < 0.001). rTregs declined (*F* (2.00, 91.41) = 113.34, *p* < 0.001), while mTreg increased (*F* (1.64, 78.88) = 38.01, *p* < 0.001). CD4^−^CD8^−^ lymphocytes decreased over time (*F* (2.00, 96.00) = 4.28, *p* = 0.017). Among inflammatory markers, GDF‐15 (*F* (2.00, 96.00) = 6.30, *p* = 0.004) and IL‐1ra increased over time (*F* (2.00, 96.00) = 3.75, *p* = 0.023).

### 3.1 Group Effects by Gender and CMV Status on T Cell Subsets

**FIGURE 1:**
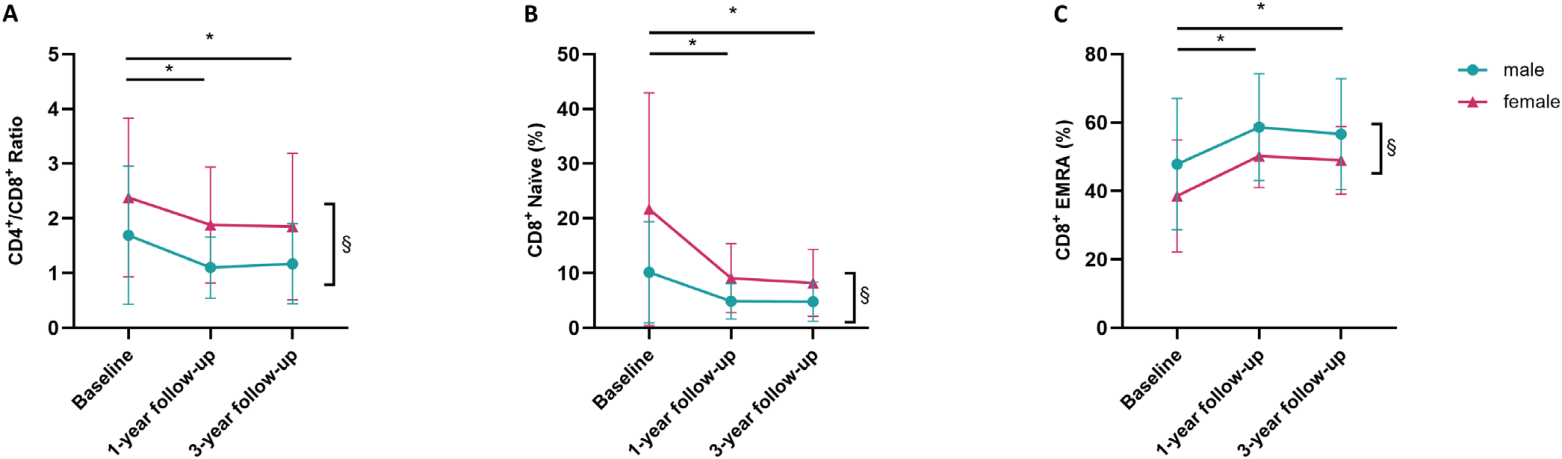
(A) Stratified analyses by gender on CD4^+^/CD8^+^ Ratio. (B) Stratified analyses by gender on CD8^+^ naïve T cells. (C) Stratified analyses by gender on CD8^+^ effector memory T cells re‐expressing CD45RA (EMRA). * indicates post hoc differences of time point *p* < 0.05.

Stratified analyses revealed distinct effects of sex on selected T cell parameters (Figure 1). Women exhibited a higher CD4^+^/CD8^+^ ratio than men (*F* (1, 47) = 9.03, *p* = 0.004; Figure 1A) and higher frequencies of naïve CD8^+^ T cells (*F* (1.00, 47.00) = 10.30, *p* = 0.002; Figure 1B). In contrast, CD8^+^ EMRA T cells were less abundant in women (*F* (1.00, 47.00) = 9.03, *p* = 0.010; Figure 1C). No additional T cell subsets showed significant sex‐dependent effects (Tables S1 and S2).

**FIGURE 2:**
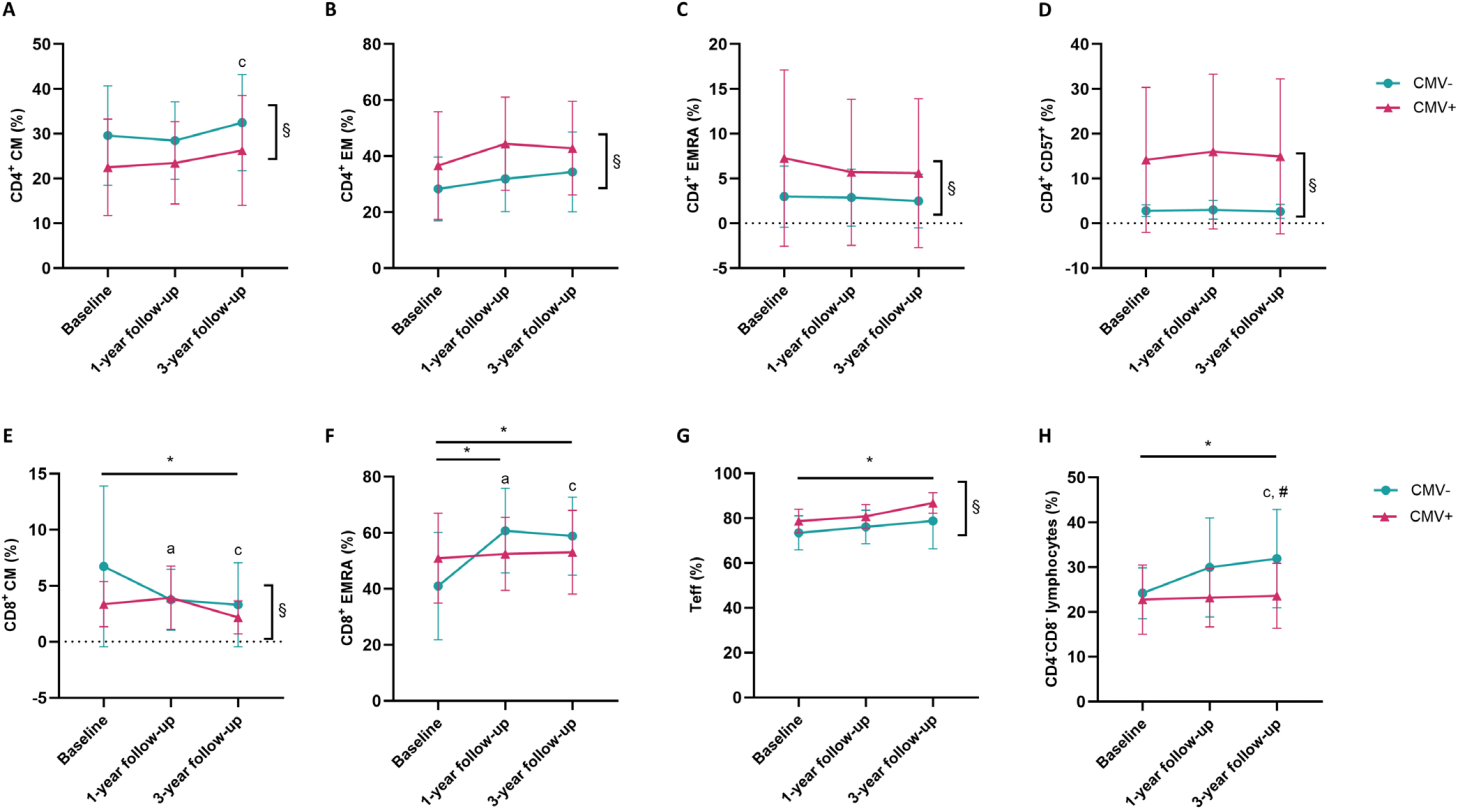
Stratified analyses by cytomegalovirus serostatus (CMV) (A) on CD4^+^ Central Memory (CM) T cells, (B) on CD4^+^ Effector Memory (EM), (C) on CD4^+^ effector memory T cells re‐expressing CD45RA (EMRA), (D) on CD4^+^CD57^+^ T cells, (E) CD8+ CM T cells, (F) on CD8^+^ EMRA T cells, (G) Effector T cells (Teff) and (H) on CD4^−^CD8^−^ lymphocytes. *Indicates post hoc differences of time point; *p* < 0.05. “a” indicates post hoc differences within CMV‐negative between baseline and 1‐year follow‐up, “b” between 1‐year follow‐up and 3‐year follow‐up and “c” between baseline and 3‐year follow‐up. # indicates post hoc difference between group and time point (interaction); *p* < 0.05. § indicates differences between groups; *p* < 0.05.

CMV serostatus was associated with pronounced differences in both CD4^+^ and CD8^+^ T cell compartments (Figure 2). CMV‐negative individuals displayed higher frequencies of CD4^+^ CM T cells (*F* (1.00, 46.00) = 5.46, *p* = 0.024; Figure 2A), whereas CMV‐positive individuals exhibited higher proportions of CD4^+^ EM (*F* (1.00, 46.00) = 8.41, *p* = 0.006; Figure 2B), CD4^+^ EMRA (*F* (1.00, 46.00) = 4.13, *p* = 0.048; Figure 2C), and CD57^+^ T cells (*F* (1.00, 46.00) = 14.41, *p* < 0.001; Figure 2D). Within the CD8^+^ compartment, CMV‐negative individuals showed higher CM T cell frequencies (*F* (1.00, 46.00) = 4.65, *p* = 0.036) with a significant group‐by‐time interaction (*F* (1.34, 61.50) = 5.49, *p* = 0.014; Figure 2E). A significant group‐by‐time interaction was also observed for CD8^+^ EMRA T cells (*F* (1.53, 118.80) = 8.58, *p* < 0.001; Figure 2F), indicating partial convergence of EMRA frequencies between groups over follow‐up. Teff cells were more frequent in CMV‐positive individuals (*F* (1.00, 46.00) = 11.11, *p* = 0.002; Figure 2G). Conversely, CD4^−^CD8^−^ lymphocytes showed higher frequencies in CMV‐negative individuals (*F* (1.00, 46.00) = 7.46, *p* = 0.009) and showed a group‐by‐time interaction (*F* (1.98, 91.27) = 3.47, *p* = 0.036; Figure 2H). Full datasets are provided in Tables S3 and S4.

### 3.2 Correlation Analyses

**FIGURE 3:**
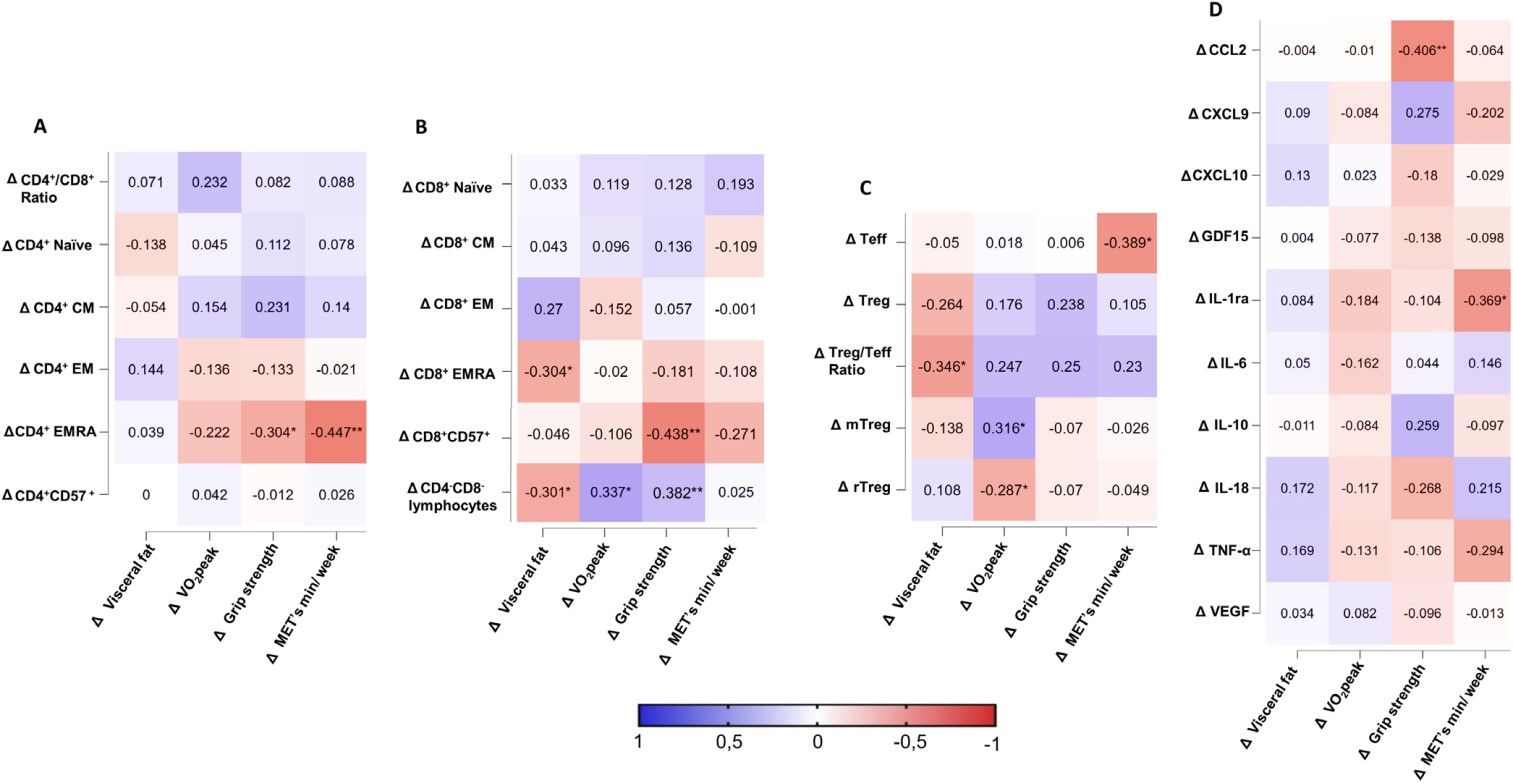
Heatmaps of correlations between 3‐year changes in physical performance, physical activity, and alterations in (A) ΔCD4 + CD8+ Ratio and CD4^+^ T cell subsets, (B) in CD8^+^ T cell subsets and CD4^−^CD8^−^ lymphocytes, (C) Teff and Tregs as well as their subpopulations and (D) in serum cytokine levels in healthy older adults. *p < 0.05, **p < 0.01. Δ indicates change over the 3‐year period (follow‐up minus baseline). BMI, body mass index; CCL, C‐C motif chemokine ligand; CD, cluster of differentiation; CM, central memory T cells; CXCL, C‐X‐C motif chemokine ligand; EM, effector memory T cells; EMRA, effector memory T cells re‐expressing CD45RA; GDF, growth differentiation factor; IL, interleukin; IL‐1ra, interleukin‐1 receptor antagonist; MET, metabolic equivalent of task; Nm, Newton meter; Teff, T effector cells; Treg, regulatory T cells; rTreg, naïve Treg cells; mTreg, memory Treg cells; TNF‐α, tumor necrosis factor‐alpha; VEGF, vascular endothelial growth factor; VO_2peak_, peak oxygen uptake.

Correlation analyses based on individual differences (Δ = 3‐year follow‐up minus baseline) revealed consistent associations between changes in physical performance and immunological parameters (Figure 3). ΔCD4^+^ EMRA T cells were negatively correlated with changes in weekly physical activity (*p* = 0.005) and grip strength (*p* = 0.040; Figure 3A). ΔCD8^+^ EMRA T cells were inversely associated with Δvisceral fat mass (*p* = 0.034), and ΔCD8^+^CD57^+^ T cells were inversely correlated with Δgrip strength (*p* = 0.002; Figure 3B). Changes in VO_2peak_ (*p* = 0.007) and grip strength (p = 0.009) were positively correlated with ΔCD4^−^CD8^−^ lymphocytes (Figure 3B). In addition, ΔTeff was negatively correlated with Δphysical activity (*p* = 0.005), whereas changes in visceral fat mass were negatively correlated with ΔTreg/Teff ratio (*p* = 0.019; Figure 3C). ΔVO_2peak_ was positively correlated with changes in mTreg (*p* = 0.005) and inversely correlated with changes in rTreg (*p* = 0.047; Figure 3C). Regarding cytokines, Δgrip strength was negatively correlated with ΔCCL2 (*p* = 0.005) and Δphysical activity was negatively correlated with ΔIL‐1ra (*p* = 0.023; Figure 3D). No additional significant correlations were observed.

### 3.3 Stepwise Linear Regression Models

To validate the associations identified in the correlation analyses, hierarchical stepwise linear regression models were applied. The most consistent and robust associations are presented in Table 2, with additional findings reported in Tables S5–S7. Changes in VO_2peak_ were significantly associated with changes in CD4^−^CD8^−^ lymphocytes after adjustment for age, sex, CMV serostatus, and changes in visceral fat, explaining up to 20% of the variance in the fully adjusted model (*p* = 0.005). CMV seropositivity additionally contributed to this model (*p* = 0.018; Table 2). VO_2peak_ changes were also associated with regulatory T cell dynamics, including positive associations with mTreg (*p* = 0.033) and inverse associations with rTreg (*p* = 0.047), although these relationships were attenuated after adjustment for sex and CMV status (Table S5). Preservation of grip strength independently predicted increases in CD4^−^CD8^−^ lymphocyte frequencies across all regression models, explaining up to 26% of the variance in the fully adjusted model (*p* = 0.002; Table 2). Further exploratory analyses revealed additional, albeit modest, associations between changes in grip strength and selected immunological parameters (Table S6). A greater decline in grip strength was associated with an increase in CD4^+^ EMRA T cells in the unadjusted model only (*p* = 0.049), while this relationship was no longer evident after covariate adjustment. A comparable pattern was observed for CD8^+^CD57^+^ T cells, with a significant association persisting up to Model 3 (*p* = 0.045), but attenuating in the fully adjusted model. Moreover, changes in grip strength were associated with alterations in circulating CCL2 levels in three of five regression models (Table S6). Changes in physical activity levels showed consistent associations with immune parameters. Specifically, changes in metabolic equivalent minutes (ΔMETs) were robustly associated with changes in effector T cell (ΔTeff) frequencies across all regression models. In the fully adjusted model, ΔMETs remained a significant independent predictor, explaining 19% of the variance (*p* < 0.001; Table 2). In addition, ΔMETs was inversely associated with changes in CD4^+^ EMRA T cells across all models (*p* = 0.016; Table S7). By contrast, the association between ΔMETs and changes in IL‐1ra was restricted to the unadjusted model and did not persist after covariate adjustment (*p* = 0.045; Table S7).

**TABLE 2:** Hierarchical stepwise linear regression models assessing the association between changes in measurements.

ΔCD4^−^CD8^−^ lymphocytes	Model 1	Model 2	Model 3	Model 4	Model 5
	ß (95% CI)	ß (95% CI)	ß (95% CI)	ß (95% CI)	ß (95% CI)
ΔVO_2peak_	0.84 (0.24 to 1.44)**	0.80 (0.17 to 1.43)**	0.86 (0.21 to 1.51)*	0.98 (0.35 to 1.62)**	0.93 (0.29 to 1.57)**
Age (baseline)	—	−0.15 (−0.84 to 0.54)	−0.10 (−0.80 to 0.61)	−0.15 (−0.84 to 0.53)	−0.03 (−0.74 to 0.68)
Sex (female)	—	—	−2.10 (−7.42 to 3.22)	−1.30 (−6.53 to 3.92)	−1.73 (−6.96 to 3.51)
CMV‐positive	—	—	—	−5.72 (−10.70 to −0.74)*	−6.08 (−11.07 to −1.09)*
ΔVisceral fat (%)	—	—	—	—	−0.46 (−1.20 to 0.29)
Adjusted R^2^	0.13**	0.11*	0.10*	0.19*	0.20*

ΔCD4^−^CD8^−^ lymphocytes	ß (95% CI)	ß (95% CI)	ß (95% CI)	ß (95% CI)	ß (95% CI)
Δgrip strength	0.87 (3.25 to 12.46)**	0.83 (0.18 to 1.48)*	0.84 (0.17 to 1.51)*	1.05 (0.41 to 1.69)**	1.03 (0.40 to 1.67)**
Age (baseline)	—	−0.29 (−0.97 to 0.39)	−0.30 (−0.98 to 0.39)	−0.36 (−1.01 to 0.29)	−0.21 (−0.88 to 0.46)
Sex (f)	—	—	0.24 (−5.14 to 5.63)	1.91 (−3.30 to 7.11)	1.11 (−4.14 to 6.36)
CMV‐ positive	—	—	—	−7.22 (−12.26 to −2.17)**	−7.47 (−12.45 to −2.48)**
ΔVisceral fat (%)	—	—	—	—	−0.54 (−1.28 to 0.20)
Adjusted *R* ^2^	0.13**	0.12	0.10	0.24**	0.26**

ΔTeff	ß (95% CI)	ß (95% CI)	ß (95% CI)	ß (95% CI)	ß (95% CI)
ΔMET's min/week	−0.001 (−0.002 to −3.61 × 10^−4^)**	−0.001 (−0.002 to −4.73 × 10^−4^)**	−0.001 (−0.002 to −4.57 × 10^−4^)**	−0.001 (−0.002 to −6.57 × 10^−4^)***	−0.001 (−0.002 to −6.38 × 10^−4^)***
Age (baseline)	—	−0.32 (−0.82 to 0.18)	−0.33 (−0.84 to 0.19)	−0.31 (−0.82 to 0.21)	−0.30 (−0.86 to 0.25)
Sex (f)	—	—	0.25 (−3.78 to 4.27)	0.53 (−4.64 to 3.58)	0.54 (−4.72 to 3.65)
CMV‐positive	—	—	—	−2.45 (−6.30 to 1.40)	−2.45 (−6.37 to 1.46)
ΔVisceral fat (%)	—	—	—	—	0.02 (−0.63 to 0.60)
Adjusted *R* ^2^	0.18**	0.19**	0.17*	0.22*	0.19*

*Note:* Standardized regression coefficients (β) with 95% confidence intervals (CI) are shown. Δ indicates change over the 3‐year period (follow‐up minus baseline). Adjusted *R* ^2^ represents variance explained by the model.

Abbreviations: CMV, cytomegalovirus serostatus; MET, metabolic equivalent of task; Teff, T effector cells; VO_2peak_, peak oxygen uptake.

* *p* < 0.05.

** *p* < 0.01.

*** *p* < 0.001.

## 4 Discussion

This longitudinal study supports the concept that 3‐year fitness trajectories are closely linked to immunological aging in healthy older adults. While most prior work has primarily emphasized cross‐sectional associations between exercise and immune function, our findings extend this knowledge by showing that intraindividual changes in aerobic capacity and muscular strength predict shifts in immune cell populations particularly within the regulatory and senescent compartments. Even in clinically healthy aging, modest changes in fitness and strength were consistently associated with immune remodeling. The results demonstrate a clear shift toward an “aged” T cell profile, characterized by reduced naïve and regulatory populations alongside increased late/terminally differentiated CD4^+^ and senescent‐associated CD8^+^ subsets, thereby altering the effector–regulatory balance. Beyond these established T cell dynamics, we also observed an increase in CD4^−^CD8^−^ lymphocytes in individuals with declining fitness. Given that this population represents a heterogeneous pool including B and NK cells, their role in the context of fitness‐related immune adaptation remains unclear and warrants further investigation. Collectively, our findings reinforce that immune aging is not a fixed process but rather a plastic trajectory that can be modulated by behavioral factors. Moreover, they fill a gap in the literature, as longitudinal data spanning multiple years in well‐characterized elderly cohorts remain scarce compared with cross‐sectional studies or short‐term interventions.

Our longitudinal data reveal a coherent trajectory of physical and immunological aging, characterized by selective declines and compensatory shifts. While overall body weight and reported activity levels remained stable, body composition displayed dynamic alterations, with a biphasic pattern in adiposity showing initial reductions followed by rebounds in body fat and visceral fat (Rosendo‐Silva et al. 2024). These changes coincided with a continuous deterioration in physical performance, particularly in VO_2peak_ and upper as well as lower limb strength, indicating an insidious loss of functional capacity despite preserved activity behavior (Baker et al. 2018). This apparent dissociation between stable self‐reported activity and declining physiological function may reflect age‐related reductions in training responsiveness, anabolic resistance, or neuromuscular efficiency (Jankowski et al. 2008), underlining the importance of distinguishing between activity behavior and functional fitness in aging research. Importantly, this dissociation provides novel longitudinal findings that immune aging trajectories are more closely aligned with changes in objectively measured functional capacity than with self‐reported activity behavior. This distinction cannot be resolved in cross‐sectional designs and highlights physical fitness as a biologically more relevant determinant of immune aging than behavioral exposure alone. At a cellular level, we observed a classic signature of immune aging within just 3 years, including reductions in naïve CD4^+^ and CD8^+^ T cells along with an expansion of memory and terminally differentiated subsets such as CD8^+^ EMRA and CD57^+^ cells (Li et al. 2019). The concomitant increase in effector T cells and reduction in Treg frequencies, particularly in rTreg, suggests a progressive loss of immune homeostasis. Notably, the decline in Tregs and the resulting drop in the Treg/Teff ratio may blunt control of excessive immune activation, a hallmark of inflammaging and chronic degenerative disease (Böttrich et al. 2023; Rocamora‐Reverte et al. 2020). The decline in the CD4^+^/CD8^+^ ratio is consistent with an “immune risk profile” (Wikby et al. 2008). In contrast, the inflammatory profile in serum remained largely stable. Apart from elevations in GDF‐15 and IL‐1ra, we did not observe consistent increases in classic pro‐inflammatory cytokines such as IL‐6 or TNF‐α. The rise in GDF‐15, a stress‐responsive cytokine secreted under mitochondrial stress, oxidative stress, or cellular senescence, and the concurrent increase in IL‐1ra, a compensatory antagonist dampening IL‐1–driven inflammation, suggest a shift toward stress‐related and regulatory signaling rather than overt inflammation (Reyes and Yap 2023). Importantly, this pattern aligns with the concept of healthy aging, in which subtle compensatory adaptations emerge in parallel with cellular immune remodeling, but frank systemic inflammation is not yet manifest. This pattern suggests that cellular immune remodeling precedes overt inflammaging and may represent an early, subclinical phase of immune aging characterized primarily by shifts in cellular composition rather than systemic cytokine reaction. Such temporal uncoupling between immune cell aging and circulating inflammatory markers has not been captured in prior cross‐sectional studies. Together, these findings suggest that distinct layers of immune aging may unfold asynchronously while cellular immune remodeling is already detectable within a short time frame; systemic inflammation appears to remain buffered through compensatory mechanisms. This temporal dissociation may provide a valuable framework for understanding how trajectories of physical fitness intersect with immune aging.

Stratified analyses highlighted distinct modulatory influences of gender and latent CMV infection, two well‐established but often underappreciated drivers of immune heterogeneity in aging. Women consistently exhibited higher CD4^+^/CD8^+^ ratios and preserved frequencies of naïve CD8^+^ T cells as well as fewer CD8^+^ EMRA T cells across all time points, aligning with prior observations of a more youthful immune profile and slower immunosenescence trajectory in females (Campbell and Turner 2018; Muller et al. 2015). These differences may reflect both hormonal influences and genetic factors, such as X‐linked immune gene expression, and underscore the need for sex‐specific reference frameworks in immunological aging research (Gubbels Bupp et al. 2018). By contrast, CMV seropositivity emerged as a potent modifier of T cell composition, particularly within CD4^+^ and the CD8^+^ compartment. CMV‐positive individuals displayed persistently elevated levels of EM and EMRA T cells, hallmarks of chronic antigenic stimulation, while showing blunted dynamics in central memory T cells over time (Hassouneh et al. 2021). These patterns are consistent with a CMV‐driven “memory inflation,” wherein long‐term viral latency imposes a cumulative cost on immune diversity and homeostasis. Remarkably, in CMV‐negative individuals, CD8^+^ EMRA T cells increased sharply during the follow‐up period, effectively closing the initial gap between groups (Cicin‐Sain 2019). This suggests that even in the absence of overt viral reactivation, age‐related immune remodeling may converge toward a similar end phenotype potentially accelerated by latent infections, reduced fitness, or systemic stress exposure.

Correlations and the regression analyses further reinforced the link between functional decline and immunological remodeling, supporting a multidimensional concept of physical–immune aging. Notably, reductions in VO_2peak_ and grip strength, two widely accepted biomarkers of functional aging (Kemala Sari et al. 2025; Letnes et al. 2023) emerged as predictors of these compositional shifts. Losses in grip strength and reductions in habitual physical activity were consistently associated with more differentiated senescent T cell phenotypes (e.g., CD4^+^ EMRA as well as CD57^+^ T cells), consistent with a previous study (Kunutsor et al. 2021). These subsets have been implicated in pro‐inflammatory signaling, impaired proliferative capacity, and diminished responsiveness to novel antigens (Callender et al. 2018), potentially amplifying the low‐grade chronic inflammation characteristics of immunosenescence (Pangrazzi and Weinberger 2020). The inverse association with functional capacity suggests that maintenance of muscle strength may act as a biological buffer against the expansion of senescent immune phenotypes, potentially by reducing inflammatory tone, metabolic stress, or oxidative load (Englund et al. 2021). Interestingly, habitual physical activity levels remained stable across the study period, yet even subtle individual changes in activity (METs/week) were significantly associated with alterations in Teff and CD4^+^ EMRA cells, emphasizing the biological relevance of intraindividual variability rather than group‐level trends. This indicates that habitual physical activity captures a behavioral dimension of immune aging that is partly independent of longitudinal changes in physiological fitness.

Moreover, distinct patterns were noted in regulatory T cell subsets. The divergent associations of rTregs and mTregs with changes in VO_2peak_ point toward differential adaptive mechanisms within the regulatory compartment. Specifically, the negative correlation between ΔrTregs and ΔVO_2peak_ suggests that individuals who maintained or improved aerobic capacity exhibited a stronger decline in rTregs, whereas greater fitness loss was accompanied by relative preservation or increases in this subset. In contrast, the positive correlation between ΔmTregs and ΔVO_2peak_ indicates that improvements in cardiorespiratory fitness were accompanied by increases in mTregs. While not all associations remained significant after full adjustment, the consistent directionality suggests a shift from thymically derived to peripherally induced regulatory phenotypes, which may represent a compensatory but potentially less stable anti‐inflammatory mechanism (Zi et al. 2025). This pattern is in line with our previous cross‐sectional observations in the same cohort, where VO_2peak_ was independently associated with higher mTreg frequencies, but inversely associated with rTregs, while studies that considered total Tregs without subpopulation‐specific differentiation reported only global increases with fitness (Böttrich et al. 2023). As we argued previously, regular physical activity can act as a pro‐inflammatory stimulus related to stress‐induced immune responses or exercise‐induced muscle damage, thereby inducing regulatory countermeasures. Under such conditions, Tregs may differentiate into memory‐like subtypes with effector functions, facilitating resolution of inflammation and supporting tissue repair. Conversely, when fitness declines, the pro‐inflammatory challenge elicited by regular activity diminishes, and the demand for memory‐like regulatory T cells may be reduced. In this context, rTregs may persist or even expand, while mTregs are no longer preferentially maintained, reflecting an adaptation of the regulatory repertoire to the functional demands placed on the immune system. Our longitudinal findings thus extend previous cross‐sectional evidence by showing that dynamic changes in fitness are accompanied by a shift in the balance between rTregs and mTregs, highlighting the plasticity of immunoregulatory capacity in aging. This dynamic balance between thymically derived and peripherally induced Tregs may reflect both immunological and metabolic adaptation to systemic stress. Notably, these findings extend beyond quantitative changes in total regulatory T cells by demonstrating a qualitative reorganization within the regulatory compartment itself. The opposing trajectories of thymically derived and peripherally induced regulatory subsets reveal a level of immunoregulatory adaptation that would remain concealed in analyses lacking longitudinal and subset‐specific resolution.

Both VO_2peak_ and grip strength were associated with changes in CD4^−^CD8^−^ lymphocytes, a heterogeneous population whose functional relevance in the context of aging and fitness is poorly defined. In our analysis, the associations persisted after controlling for covariates; however, given the mixed cellular composition of this population, the finding should primarily be viewed as exploratory. It remains unclear whether the observed increase reflects compensatory adaptation, unspecific immune remodeling, or other mechanisms related to declining physical capacity. Further studies using refined phenotyping and functional assays are needed to delineate the underlying cell populations and their physiological significance. Notably, the CD4^−^CD8^−^ lymphocyte compartment likely encompasses NK cells and innate‐like T cell subsets such as MAIT and γδ T cells. In line with this interpretation, our previous work in a subset of this cohort demonstrated that endurance‐trained older adults exhibit enhanced NK‐cell metabolic flexibility and effector regulation under adrenergic and mTOR (Minuzzi et al. 2025). Together, these findings suggest that the preservation of innate immune metabolism through regular exercise may contribute to maintaining immune plasticity and resilience in aging. Importantly, CMV seropositivity emerged as a consistent negative predictor, underscoring its established role as a chronic modifier of immune plasticity across functional gradients (Poloni et al. 2022).

Adiposity and inflammation‐related markers added further granularity to this functional‐immune interplay. Visceral fat gain was linked to increases in CD8^+^ EMRA cells and reductions in the Treg/Teff ratio, the known immunometabolic crosstalk that links central adiposity to chronic low‐grade inflammation and immune dysregulation (Gálvez et al. 2023; Li et al. 2020). In line with this, declines in physical activity were associated with elevated IL‐1ra levels, and reduced grip strength correlated with higher CCL2, two cytokines implicated in monocyte recruitment, tissue remodeling, and age‐related disease progression (Cesari et al. 2004). Although the explained variance of some models remained modest, the reproducibility of associations across fully adjusted models supports a biologically meaningful link between physical function and immune cell remodeling in older adults.

Several limitations must be acknowledged when interpreting the present findings. First, the study lacks a control group of younger individuals, which limits conclusions regarding age‐related specificity of the observed immune trajectories. The interpretation of immunosenescence markers and their modulation through lifestyle factors would benefit from a direct age comparison. Second, the observational design of the study precludes causal inference. While several associations between physical fitness, body composition, and immune parameters were identified, no conclusions can be drawn about the directionality or underlying mechanisms of these relationships. Interventional studies are needed to test whether modifying fitness or body composition leads to changes in immune aging. Third, habitual physical activity was assessed using a self‐reported questionnaire rather than objective monitoring. Consequently, conclusions regarding behavioral stability or training responsiveness rely on subjective reporting and may be influenced by recall bias or misclassification. This limitation is particularly relevant in older adults, where perceived activity levels may not accurately reflect physiological load. Notably, the dissociation between stable self‐reported activity and declining cardiorespiratory fitness and strength highlights the importance of distinguishing reported behavior from objectively measured functional capacity. Fourth, the sample size is relatively small, which may reduce the statistical power. As a result, potentially relevant associations might have remained undetected. In addition, potential selection bias must be considered. Participants were physically healthy older adults who voluntarily participated in a longitudinal study, possibly leading to an overrepresentation of health‐conscious and resilient individuals. This may limit the generalizability of the findings to the broader aging population. To minimize the influence of missing data, only participants with complete datasets at all baseline and follow‐up measurements were included in the analysis. While this approach ensured consistency in the longitudinal analyses, it may have led to the selective inclusion of more robust or adherent individuals, potentially introducing subtle biases in the sample composition. Fifth, although all participants were clinically healthy and free from chronic disease, subclinical biological processes cannot be entirely excluded. Previous analyses in the same cohort indicated that subtle physiological remodeling may occur even in the absence of overt pathology, reflecting normal variation within the biological aging process. Such subclinical differences could have contributed to interindividual variability in immune trajectories observed here. Lastly, the sample shows an imbalance in sex distribution, which may have influenced immune parameters known to differ between males and females. Although sex was controlled for in analyses, residual confounding cannot be excluded.

In conclusion, our findings highlight a multidimensional interplay between physical fitness and immune remodeling during aging. Using a longitudinal, intraindividual design, this study moves beyond predominantly cross‐sectional evidence by demonstrating that trajectories of objectively measured physical fitness and muscular strength are closely linked to dynamic changes in immune cell composition over a 3‐year period. Even in the context of clinically healthy aging, modest but consistent declines in cardiorespiratory fitness and strength were associated with a shift toward an aged immune phenotype, characterized by effector and senescent cell expansion, loss of naïve and regulatory T cell populations, and imbalances within regulatory T cell subsets. Associations with CD4^−^CD8^−^ lymphocytes further point to immune adaptations extending beyond conventional T cell aging and warrant refined phenotypic and functional investigation. Importantly, our data reveal that immune remodeling can occur independently of overt disease and largely in the absence of classical systemic inflammaging, suggesting an early and subclinical stage of immune aging dominated by cellular reorganization. The observed dissociation between stable self‐reported physical activity and declining functional capacity indicates that immune aging trajectories are more closely aligned with changes in physiological fitness than with behavioral exposure alone. Notably, habitual physical activity, despite remaining stable at the group level, was independently associated with effector T cell dynamics, underscoring the relevance of behavioral variability as a complementary layer of immune aging. By capturing immune remodeling in clinically healthy older adults before the onset of overt pathology, this study characterizes immune aging as a progressive and subclinical process. Together, these findings indicate that immune aging unfolds as a dynamic process that emerges early in healthy aging and remains sensitive to both physiological fitness and habitual activity, identifying physical fitness as a primary and physical activity as a complementary determinant of immune trajectories and potential targets for preserving immune resilience.

## Author Contributions

Christopher Weyh: Conceptualization, methodology, validation, investigation, writing – original draft, writing – review and editing, visualization, and supervision. Vincent Größer: Methodology and investigation. Luciele Guerra Minuzzi: Writing – original draft and writing – review and editing. Torsten Frech: Investigation. Kristina Gebhardt: Investigation. Svenja Nolte: Investigation. Theresa R. Dombrowski: Investigation. Manuela Andrea Hoffmann: Writing – review and editing. Natascha Sommer: Conceptualization, methodology, and funding acquisition. Robert Ringseis: Conceptualization, methodology, validation, investigation, writing – review and editing, and funding acquisition. Klaus Eder: Conceptualization, writing – review and editing, and funding acquisition. Samuel Sossalla: Methodology, writing – review and editing. Pascal Bauer: Conceptualization, methodology, validation, investigation and funding acquisition. Karsten Krüger: Conceptualization, methodology, writing – original draft, writing – review and editing, supervision, and funding acquisition.

## Funding

This research was funded by the Forschungscampus Mittelhessen, Flexi Fund Project number 20121_1_1_10.

## Ethics Statement

The study protocol was approved by the Ethics Committee of the Justus Liebig University Giessen (Ref. No. 100/20), and all procedures were conducted in accordance with the Declaration of Helsinki. Written informed consent was obtained from all participants prior to enrollment. A clinical trial registration number is not applicable due to the non‐interventional design of this observational study.

## Conflicts of Interest

The authors declare no conflicts of interest.

## Supporting information


**Appendix S1:** acel70440‐sup‐0001‐AppendixS1.docx.

## Data Availability

The raw data supporting the conclusions of this article will be made available by the authors, without undue reservation.
