# Supplementary material for: Physical Fitness Dynamics Shape Immune Remodeling in Healthy Aging: A 3‐Year Longitudinal Study
Source: Aging Cell. 2026 Mar 6;25(3):e70440. doi: 10.1111/acel.70440 (PMC12965826; doi:10.1111/acel.70440)
Supplement: Supplementary file 1 — Appendix S1: acel70440‐sup‐0001‐AppendixS1.docx. [file ACEL-25-e70440-s001.docx]

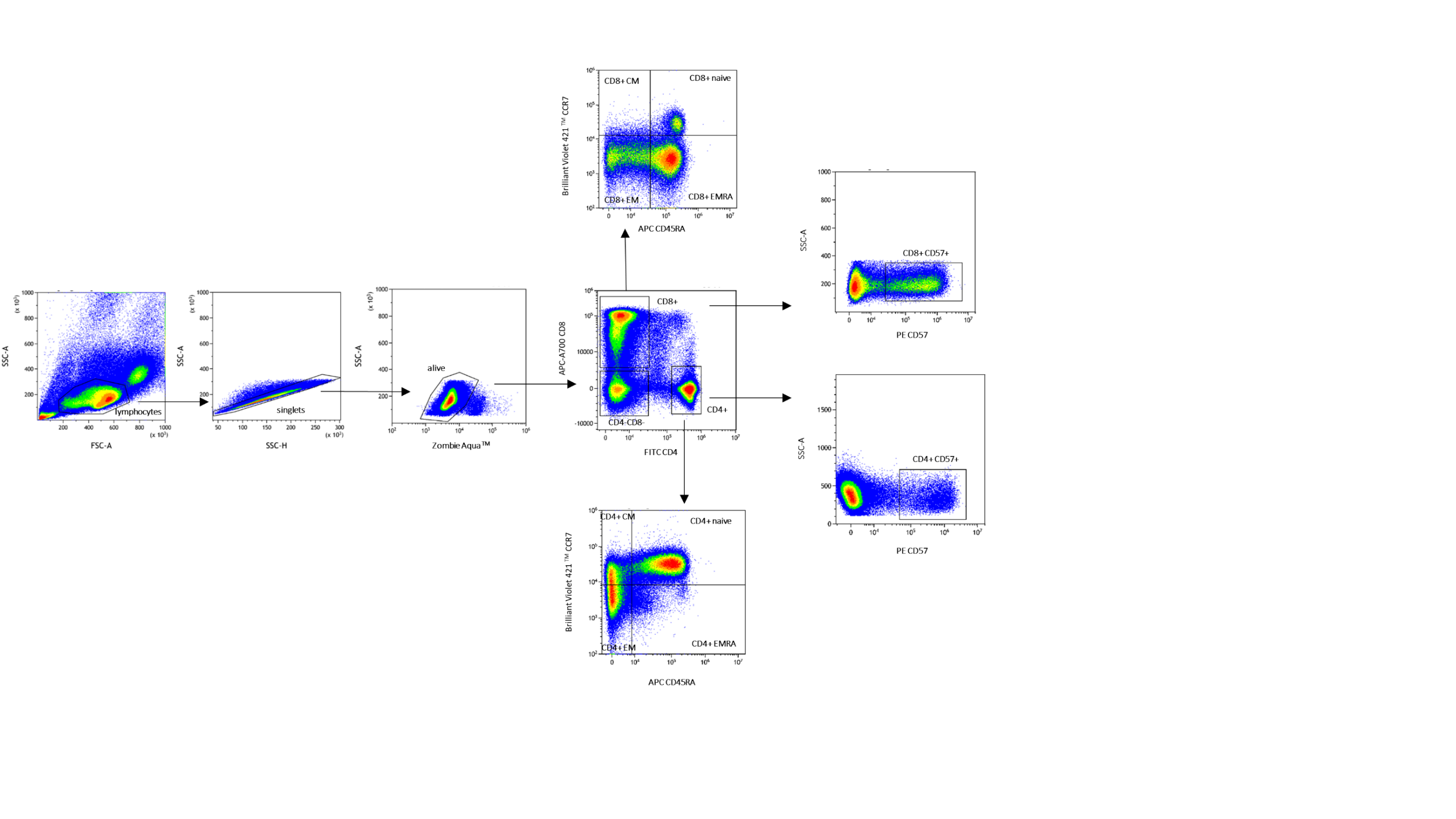


Figure S1. Frozen PBMCs were thawed and stained with fluorochrome-conjugated antibodies. Gating strategy: Lymphocytes were gated in a FSC/SSC-dot plot and dead cells excluded using Zombie Aqua^TM^. From the living cell population, CD4^+^ and CD8^+^T-cells as well as CD4^-^CD8^-^ lymphocytes, were gated according. T cell subsets were gated in CD4+ and CD8+ T cells as naïve T cells (CCR7^+^CD45RA^+^), Central Memory T cells (CCR7^+^CD45RA^-^), Effector Memory T cells (CCR7^-^CD45RA^-^) and effector memory T cells re-expressing CD45RA (EMRA) (CCR7^-^CD45RA^+^). Additionally, CD57^+^ were gated on CD4^+^ and CD8^+^ T cells as CD4^+^CD57^+^ and CD8^+^CD57^+^.


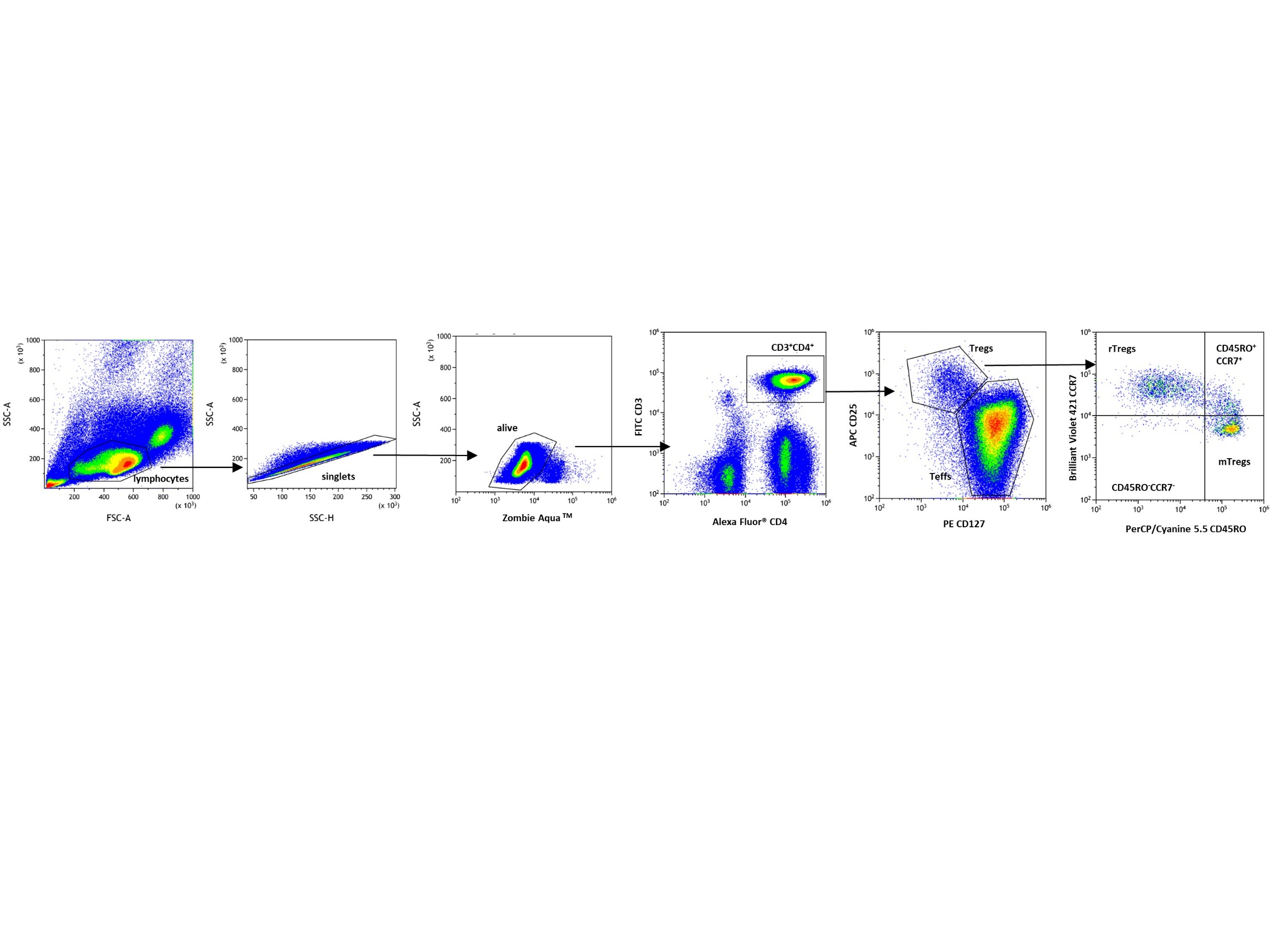


Figure S2. Frozen PBMCs were thawed and stained with fluorochrome-conjugated antibodies. Gating strategy: Lymphocytes were gated in a FSC/SSC-dot plot and dead cells excluded using Zombie Aqua^TM^. From the living cell population, CD4 T cells, were gated according to CD3^+^/CD4^+^ (A). T effector cells (Teff) were gated as CD4^+^CD25^+^CD127^high^. T_regs_ were defined and gated as CD4^+^CD25^+^CD127^low^. Subsets of Tregs were gated as rTregs(CD45RO^-^CCR7^+^) and mTregs(CD45RO^+^CCR7^-^).

Table S1. Longitudinal changes in T cell subset distributions and regulatory/effector balance activity in healthy older adults over a 3-year follow-up stratified by sex.

|  | Baseline | | 1-Year Follow-Up | | 3-Year Follow-Up | | Time  p-value | Group  p- value | Time x group  p-value |
| --- | --- | --- | --- | --- | --- | --- | --- | --- | --- |
|  | Male | female | male | female | male | female |  |  |  |
| CD4^+^/CD8^+^ Ratio | 1.7 ± 1.3 | 2.4 ± 1.5 | 1.1 ± 0.6 | 1.9 ± 1.1 | 1.2 ± 0.7 | 1.85 ± 1.34 | **0.003^a,c^** | **0.004** | 0.951 |
| CD4^+^ Naïve (%) | 33.8 ± 15.4 | 40.6 ± 18.6 | 28.5 ± 14.2 | 35.0 ± 17.0 | 26.5 ± 13.6 | 29.6 ± 18.6 | **≤0.001^a,c^** | 0.167 | 0.663 |
| CD4^+^ CM (%) | 26.4 ± 11.3 | 24.4 ± 11.6 | 25.3 ± 8.1 | 26.2 ± 10.8 | 28.9 ± 10.6 | 29.2 ± 13.8 | **0.016^b,c^** | 0.926 | 0.534 |
| CD4^+^ EM (%) | 35.0 ± 15.9 | 29.2 ± 16.8 | 41.1 ± 14.6 | 33.8 ± 15.7 | 39.91± 12.7 | 36.7 ± 20.4 | **0.039** | 0.142 | 0.676 |
| CD4^+^ EMRA (%) | 4.9 ± 6.3 | 5.9 ± 9.7 | 4.3 ± 6.0 | 4.5 ± 7.1 | 4.2 ± 5.8 | 4.1 ± 7.7 | 0.347 | 0.826 | 0.793 |
| CD4^+^CD57^+^ (%) | 9.0 ± 12.7 | 8.6 ± 13.9 | 10.3 ± 14.5 | 9.3 ± 14.1 | 8.8 ± 11.7 | 9.8 ± 17.3 | 0.807 | 0.971 | 0.775 |
| CD8^+^ Naïve (%) | 10.1 ± 9.2 | 21.7 ± 21.3 | 4.8 ± 3.2 | 9. ± 6.31 | 4.8 ± 3.6 | 8.1 ± 6.1 | **≤0.001^a,c^** | **0.002** | 0.056 |
| CD8^+^ CM (%) | 4.7 ± 5.8 | 6.3 ± 7.5 | 3.0 ± 2.4 | 4.7 ± 2.6 | 2.3 ± 1.9 | 3.7 ± 3.6 | **0.015^c^** | 0.096 | 0.956 |
| CD8^+^ EM (%) | 34.2 ± 13.9 | 29.3 ± 16.4 | 32.7 ± 14.3 | 35.6 ± 10.7 | 35.8 ± 15.8 | 38.7 ± 11.4 | 0.073 | 0.920 | 0.161 |
| CD8^+^ EMRA (%) | 47.8 ± 19.2 | 38.5 ± 16.4 | 58.6 ± 15.6 | 50.2 ± 9.2 | 56.6 ± 16.2 | 49.0 ± 9.9 | **≤0.001^a,c^** | **0.010** | 0.908 |
| CD8^+^ CD57^+^ (%) | 41.0 ± 12.8 | 38.3 ± 27.3 | 44.2 ± 15.2 | 44.2 ± 14.4 | 46.3 ± 15.7 | 46.31 ± 14.96 | **0.004^c^** | 0.799 | 0.739 |
| Teff (%) | 76.0 ± 7.2 | 75.4 ± 6.8 | 78.2 ± 7.0 | 78.4 ± 6.7 | 82.1 ± 11.5 | 83.1 ± 8.2 | **≤0.001^a,b,c^** | 0.770 | 0.757 |
| Treg (%) | 6.1 ± 1.7 | 5.8 ± 1.4 | 4.2 ± 1.4 | 4.1 ± 1.5 | 2.9 ± 1.3 | 3.0 ± 1.8 | **≤0.001^a,b,c^** | 0.932 | 0.538 |
| Treg/Teff ratio | 0.08 ± 0.02 | 0.08 ± 0.02 | 0.06 ± 0.02 | 0.05 ± 0.02 | 0.03 ± 0.02 | 0.03 ± 0.02 | **≤0.001^a,b,c^** | 0.648 | 0.821 |
| rTreg (%) | 32.3 ± 8.0 | 33.5 ± 14.1 | 24.3 ± 9.9 | 20.4 ± 8.1 | 10.6 ± 7.6 | 9.5 ± 8.4 | **≤0.001^a,b,c^** | 0.553 | 0.266 |
| mTreg (%) | 33.1 ± 12.7 | 28.6 ± 13.7 | 45.1 ± 9.1 | 46.8 ± 9.0 | 52.1 ± 20.2 | 92.3 ± 19.0 | **≤0.001^a,b,c^** | 0.382 | 0.051 |
| CD4^-^CD8^-^ lymphocytes (%) | 25.2 ± 6.3 | 20.4 ± 6.6 | 27.9 ± 9.2 | 22.3 ± 8.2 | 29.3 ± 11.0 | 23.9 ± 7.2 | **0.025^c^** | 0.142 | 0.944 |

^a^ indicates post-hoc differences between baseline and 1-year follow-up, ^b^ post-hoc differences between 1-year follow-up and 3-year follow-up, ^c^ post-hoc differences between baseline and 3-year follow-up. **Abbreviations:** CD: cluster of differentiation; CM: central memory T cells; EM: effector memory T cells; EMRA: effector memory T cells re-expressing CD45RA; Teff: T effector cells; Treg: regulatory T cells; rTreg: naïve Treg cells; mTreg: memory Treg cells.

Table S2. Longitudinal changes in circulating inflammatory and regulatory cytokines parameters in healthy older adults over a 3-year follow-up stratified by sex.

|  | Baseline | | 1-Year Follow-Up | | 3-Year Follow-Up | | Time  p-value | Group  p- value | Time x group  p-value |
| --- | --- | --- | --- | --- | --- | --- | --- | --- | --- |
|  | Male | female | male | female | male | female |  |  |  |
| CCL-2 (pg/ml) | 529.5 ± 149.2 | 444.7 ± 156.8 | 538.3 ± 156.2 | 447.7 ± 173.4 | 503.8 ± 148.2 | 462.5 ± 168.0 | 0.785 | 0.100 | 0.176 |
| CXCL-9 (pg/ml) | 1054.2 ± 1593.1 | 562.9 ± 677.3 | 1048.4 ± 1666.5 | 573.8 ± 724.7 | 1054.1 ± 1608.1 | 518.2 ± 684.9 | 0.770 | 0.206 | 0.724 |
| CXCL-10 (pg/ml) | 12.9 ± 8.2 | 11.2 ± 5.8 | 14.7 ± 10.0 | 10.8 ± 4.9 | 12.5 ± 9.3 | 11.4 ± 6.2 | 0.501 | 0.312 | 0.205 |
| GDF-15 (pg/ml) | 584.0 ± 272.1 | 555.8 ± 368.8 | 637.3 ± 355.5 | 577.9 ± 403.9 | 632.7 ± 318.4 | 615.6 ± 393.0 | **0.004^a,c^** | 0.728 | 0.399 |
| IL-1ra (pg/ml) | 897.2 ± 1443.2 | 457.2 ± 151.2 | 973.5 ± 1485.8 | 507.9 ± 316.6 | 992.8 ± 1555.0 | 554.1 ± 314.0 | **0.038** | 0.201 | 0.913 |
| IL-6 (pg/ml) | 23.7 ± 47.8 | 12.0 ± 12.3 | 25.5 ± 51.3 | 11.4 ± 11.7 | 29.2 ±72.9 | 18.3 ± 35.6 | 0.188 | 0.368 | 0.747 |
| IL-10 (pg/ml) | 1.9 ± 1.5 | 1.7 ± 1.1 | 2.1 ± 1.6 | 1.9 ± 1.2 | 2.0 ± 1.5 | 1.7 ±1.6 | 0.101 | 0.547 | 0.913 |
| IL-18 (pg/ml) | 515.9 ± 297.6 | 480.8 ± 224.5 | 512.0 ± 294.3 | 480.1 ± 242.9 | 509.9 ± 309.4 | 500.5 ± 289.2 | 0.779 | 0.757 | 0.582 |
| TNF-α (pg/ml) | 7.3 ± 6.3 | 5.0 ± 3.1 | 7.5 ± 6.6 | 5.2 ± 3.0 | 7.7 ± 7.2 | 4.9 ± 2.3 | 0.860 | 0.117 | 0.740 |
| VEGF (pg/ml) | 506.4 ± 498.0 | 543.8 ± 717.0 | 562.1 ± 566.1 | 561.8 ± 696.1 | 542.5 ± 471.2 | 431.5 ± 263.2 | 0.361 | 0.867 | 0.345 |

^a^ indicates post-hoc differences between baseline and 1-year follow-up, ^b^ post-hoc differences between 1-year follow-up and 3-year follow-up, ^c^ post-hoc differences between baseline and 3-year follow-up. **Abbreviations:** CCL: C-C motif chemokine ligand; CXCL: C-X-C motif chemokine ligand; GDF: growth differentiation factor; IL: interleukin; IL-1ra: interleukin-1 receptor antagonist; TNF-α: tumor necrosis factor-alpha; VEGF: vascular endothelial growth factor.

Table S3. Longitudinal changes in T cell subset distributions and regulatory/effector balance in healthy older adults over a 3-year follow-up stratified by Cytomegalovirus serostatus (CMV).

|  | Baseline | | 1-Year Follow-Up | | 3-Year Follow-Up | | Time  p-value | Group  p- value | Time x group  p-value |
| --- | --- | --- | --- | --- | --- | --- | --- | --- | --- |
|  | **CMV-** | **CMV+** | **CMV-** | **CMV+** | **CMV-** | **CMV+** |  |  |  |
| CD4^+^/CD8^+^ Ratio | 2.4 ± 1.7 | 1.6 ± 0.8 | 1.5 ± 1.0 | 1.4 ± 0.8 | 1.6 ± 1.3 | 1.4 ± 0.9 | **<0.001^a,c^** | 0.137 | 0.066 |
| CD4^+^ Naïve (%) | 39.1 ± 18.2 | 33.6 ± 15.7 | 35.6 ± 16.0 | 26.0 ± 13.0 | 30.3 ± 17.0 | 24.6 ± 13.7 | **<0.001^a,c^** | 0.069 | 0.583 |
| CD4^+^ CM (%) | 29.6 ± 11.1 | 22.5 ± 10.7 | 28.5 ± 8.6 | 23.5 ± 9.2 | 32.5 ± 10.8 | 26.3 ± 12.3 | **0.022^b^** | **0.024** | 0.750 |
| CD4^+^ EM (%) | 28.3 ± 11.4 | 36.6 ± 19.2 | 31.9 ± 11.8 | 44.5 ± 16.6 | 34.4 ± 14.3 | 42.9 ± 16.7 | **0.033** | **0.006** | 0.593 |
| CD4^+^ EMRA (%) | 3.0 ± 3.4 | 7.3 ± 9.8 | 2.9 ± 3.2 | 5.7 ± 8.2 | 2.5 ± 3.0 | 5.6 ± 8.3 | 0.446 | **0.048** | 0.666 |
| CD4^+^CD57^+^ (%) | 2.8 ± 1.3 | 14.2 ± 16.2 | 3.0 ± 2.1 | 16.02± 17.3 | 2.7 ± 1.6 | 15.0 ± 17.3 | 0.775 | **<0.001** | 0.849 |
| CD8^+^ Naïve (%) | 17.6 ± 19.7 | 12.3 ± 12.0 | 7.1 ± 5.9 | 5.6 ± 3.9 | 7.0 ± 5.7 | 5.4 ± 4.4 | **<0.001^a,c^** | 0.200 | 0.389 |
| CD8^+^ CM (%) | 7.8 ± 8.9 | 3.3 ± 2.0 | 3.6 ± 2.5 | 3.8 ± 2.7 | 3.7 ± 3.7 | 2.2 ± 1.4 | **0.005^c^** | **0.036** | **0.014** |
| CD8^+^ EM (%) | 31.9 ± 15.7 | 31.4 ± 13.6 | 27.9 ± 12.8 | 38.1 ± 12.8 | 30.8 ± 11.2 | 39.0 ± 15.1 | 0.370 | 0.072 | 0.083 |
| CD8^+^ EMRA (%) | 37.5 ± 20.4 | 50.0 ± 15.5 | 60.0 ± 14.5 | 52.3 ± 12.3 | 55.5 ± 14.8 | 52.9 ± 14.0 | **<0.001^a,c^** | 0.830 | **<0.001** |
| CD8^+^ CD57^+^ (%) | 34.4 ± 13.8 | 43.6 ± 13.5 | 42.9 ± 15.8 | 44.9 ± 14.3 | 42.5 ± 15.3 | 48.9 ± 14.9 | **0.002^a,c^** | 0.111 | 0.168 |
| Teff (%) | 73.5 ± 7.6 | 78.7 ± 5.2 | 76.1 ± 7.5 | 80.8 ± 5.3 | 78.8 ± 12.5 | 86.8 ± 4.6 | **<0.001^a,b,c^** | **0.002** | 0.321 |
| Treg (%) | 5.6 ±1.6 | 6.3 ± 1.4 | 4.1 ± 1.5 | 4.2 ± 1.4 | 3.0 ± 1.8 | 2.8 ± 0.9 | **<0.001^a,b,c^** | 0.589 | 0.179 |
| Treg/Teff ratio | 0.08 ± 0.03 | 0.08 ± 0.02 | 0.05 ± 0.02 | 0.05 ± 0.05 | 0.04 ± 0.02 | 0.03 ± 0.01 | **<0.001^a,b,c^** | 0.704 | 0.431 |
| rTreg (%) | 30.5 ± 9.5 | 33.9 ± 9.9 | 22.7 ± 9.9 | 23.0 ± 9.1 | 10.5 ± 9.5 | 9.7 ± 5.8 | **<0.001^a,b,c^** | 0.640 | 0.312 |
| mTreg (%) | 32.7 ± 12.7 | 30.6 ± 13.6 | 46.7 ± 8.9 | 44.8 ± 9.4 | 55.4 ± 22.6 | 55.9 ± 17.5 | **<0.001^a,b,c^** | 0.684 | 0.851 |
| CD4^-^CD8^-^ lymphocytes (%) | 24.2 ± 5.7 | 22.8 ± 7.7 | 29.9 ± 11.0 | 23.2 ± 6.5 | 31.9 ± 11.0 | 23.6 ± 7.3 | **0.006^c^** | **0.009** | **0.035** |

^a^ indicates post-hoc differences between baseline and 1-year follow-up, ^b^ post-hoc differences between 1-year follow-up and 3-year follow-up, ^c^ post-hoc differences between baseline and 3-year follow-up. **Abbreviations:** CD: cluster of differentiation; CM: central memory T cells; EM: effector memory T cells; EMRA: effector memory T cells re-expressing CD45RA; Teff: T effector cells; Treg: regulatory T cells; rTreg: naïve Treg cells; mTreg: memory Treg cells.

Table S4. Longitudinal changes in circulating inflammatory and regulatory cytokines parameters in healthy older adults over a 3-year follow-up stratified by Cytomegalovirus serostatus (CMV).

|  | Baseline | | 1-Year Follow-Up | | 3-Year Follow-Up | | Time  p-value | Group  p- value | Time x group  p-value |
| --- | --- | --- | --- | --- | --- | --- | --- | --- | --- |
|  | **CMV-** | **CMV+** | **CMV-** | **CMV+** | **CMV-** | **CMV+** |  |  |  |
| CCL-2 (pg/ml) | 501.7 ± 165.8 | 484.6 ± 148.4 | 482.5 ± 169.8 | 515.3 ± 168.1 | 479.0 ± 166.0 | 486.2 ± 145.6 | 0.513 | 0.862 | 0.221 |
| CXCL-9 (pg/ml) | 754.8 ± 1519.7 | 943.0 ± 1193.8 | 725.9 ± 1441.5 | 963.2 ± 1386.8 | 703.4 ± 1291.1 | 955.2 ± 1427.1 | 0.839 | 0.570 | 0.705 |
| CXCL-10 (pg/ml) | 11.3 ± 6.1 | 13.1 ± 8.4 | 13.9 ± 8.8 | 12.7 ± 8.6 | 11.5 ± 6.6 | 12.7 ± 9.6 | 0.235 | 0.794 | 0.157 |
| GDF-15 (pg/ml) | 621.3 ± 382.7 | 532.3 ± 239.5 | 660.4 ± 450.9 | 570.1 ± 300.5 | 650.1 ± 389.1 | 602.4 ± 316.8 | **0.006^a,c^** | 0.449 | 0.318 |
| IL-1ra (pg/ml) | 847.8 ± 1217.6 | 635.8 ± 1119.0 | 901.4 ± 1304. | 722.24 ± 1129.6 | 962.4 ± 1412.9 | 721.8 ± 1119.3 | **0.026^c^** | 0.549 | 0.707 |
| IL-6 (pg/ml) | 16.9 ± 30.5 | 21.9 ± 45.1 | 17.5 ± 33.0 | 22.2 ± 48.2 | 22.3 ± 43.5 | 27.5 ± 74.5 | 0.204 | 0.728 | 0.943 |
| IL-10 (pg/ml) | 1.7 ± 1.7 | 2.0 ± 1.1 | 2.1 ± 1.3 | 2.0 ± 1.3 | 1.9 ± 1.8 | 1.9 ± 1.3 | 0.069 | 0.868 | 0.167 |
| IL-18 (pg/ml) | 538.6 ± 298.7 | 467.0 ± 248.2 | 545.3 ± 329.7 | 452.8 ± 215.0 | 542.9 ± 367.8 | 467.7 ± 231.1 | 0.874 | 0.327 | 0.678 |
| TNF-α (pg/ml) | 6.4 ± 6.4 | 6.5 ± 4.6 | 6.5 ± 6.1 | 6.6 ± 5.3 | 6.1 ± 5.6 | 7.0 ± 6.4 | 0.870 | 0.840 | 0.497 |
| VEGF (pg/ml) | 518.2 ± 712.9 | 540.9 ± 417.8 | 564.0 ± 717.5 | 578.9 ± 527.8 | 465.3 ± 436.3 | 544.0 ± 378.9 | 0.422 | 0.791 | 0.716 |

^a^ indicates post-hoc differences between baseline and 1-year follow-up, ^b^ post-hoc differences between 1-year follow-up and 3-year follow-up, ^c^ post-hoc differences between baseline and 3-year follow-up. **Abbreviations:** CCL: C-C motif chemokine ligand; CXCL: C-X-C motif chemokine ligand; GDF: growth differentiation factor; IL: interleukin; IL-1ra: interleukin-1 receptor antagonist; TNF-α: tumor necrosis factor-alpha; VEGF: vascular endothelial growth factor.

Table S5. Hierarchical linear regression models assessing the association between changes in Δ VO_2_peak and regulatory T cell subsets.

|  | Modell 1 | Modell 2 | Modell 3 | Modell 4 | Modell 5 |
| --- | --- | --- | --- | --- | --- |
| ΔmTreg | ß (95% CI) | ß (95% CI) | ß (95% CI) | ß (95% CI) | ß (95% CI) |
| ΔVO₂peak | 1.73 (0.15 – 3.30)* | 1.91 (0.26 – 3.56)* | 1.58 (-0.82 – 3.24) | 1.61 (-0.79 – 3.31) | 1.52 (-0.20 – 3.23) |
| Age (baseline) | – | 0.72 (-1.09 – 2.53) | 0.42 (-1.38 – 2.23) | 0.53 (-1.29 – 2.36) | 0.75 (-1.16 – 2.67) |
| Sex (f) | – | – | 11.83 (-1.79 – 25.45) | 11.04 (-2.89 – 24.95) | 10.30 (-3.82 – 24.41) |
| CMV-positiv | – | – | – | -5.84 (-19.13 – 7.45) | -6.48 (-19.93 – 6.96) |
| ΔVisceral fat (%) | – | – | – | – | -0.81 (-2.82 – 1.20) |
| Adjusted R² | 0.07* | 0.07 | 0.11* | 0.08 | 0.07 |
|  |  |  |  |  |  |
| ΔrTreg | β (95% CI ) | β (95% CI ) | β (95% CI ) | β (95% CI ) | β (95% CI ) |
| ΔVO₂peak | -0.77 (-1.53 – -0.01)* | -0.82 (-1.62 – -0.02)* | -0.80 (-1.62 – 0.03) | -0.80 (-1.55 – -0.04)* | -0.75 (-1.51 – 0.021) |
| Age (baseline) | – | -0.21 (-1.08 – 0.67) | -0.18 (-1.08 – 0.71) | -0.34 (-1.15 – 0.48) | -0.46 (-1.31 – 0.40) |
| Sex (f) | – | – | 0.84 (-7.62 – 5.93) | 0.37 (-5.86 – 6.61) | 0.78 (-5.52 – 7.08) |
| CMV-positiv | – | – | – | 5.22 (-0.73 – 11.17) | 5.57 (-0.42 – 11.57) |
| ΔVisceral fat (%) | – | – | – | – | -0.44 (-0.45 – 1.34)* |
| Adjusted R² | 0.06* | 0.05 | 0.03 | 0.06 | 0.06 |

**Note:** Standardized regression coefficients (β) with 95% confidence intervals (CI) are shown. Δ indicates change over the 3-year period (follow-up minus baseline). Adjusted R² represents variance explained by the model. * indicates p < 0.05, ** p < 0.01, *** p < 0.001. **Abbrevations:** CMV: cytomegalovirus serostatus; Treg: regulatory T cells; rTreg: naïve Treg cells; mTreg: memory Treg cells; VO_2peak_: peak oxygen uptake.

|  | Modell 1 | Modell 2 | Modell 3 | Modell 4 | Modell 5 |
| --- | --- | --- | --- | --- | --- |
| ΔCD4^+^ EMRA | ß (95% CI) | ß (95% CI) | ß (95% CI) | ß (95% CI) | ß (95% CI) |
| ΔHand grip | -0.21 (-0.56 – -0.02)* | -0.22 (-0.58 – 0.09) | -0.26 (-0.63 – 0.10) | -0.26 (-0.64 – 0.12) | -0.26 (-0.64 – 0.13) |
| Age (baseline) | – | -0.03 (-0.41 – 0.34) | -0.02 (-0.39 – 0.35) | -0.01 (-0.40 – 0.38) | -0.03 (-0.44 – 0.38) |
| Sex (f) | – | – | -1.09 (-4.81 – 1.01) | -1.93 (-5.02 – 1.17) | -1.83 (-5.04 – 1.37) |
| CMV-positiv | – | – | – | -0.40 (-3.40 – 2.60) | -0.37 (-3.42 – 2.67) |
| ΔVisceral fat (%) | – | – | – | – | 0.06 (-0.39 – 0.51) |
| R² | 0.03* | 0.03 | 0.05 | 0.04 | 0.04 |
|  |  |  |  |  |  |
| ΔCD8^+^CD57^+^ | ß (95% CI) | ß (95% CI) | ß (95% CI) | ß (95% CI) | ß (95% CI) |
| ΔHand grip | -0.96 (-1.79 – -0.12)* | -0.97 (-1.82 – -0.11)* | -0.90 (-1.77 – -0.02)* | -0.79 (-1.69 – 0.12) | -0.78 (-1.70 – 0.14) |
| Age (baseline) | – | -0.09 (-1.82 – 0.81) | -0.11 (-1.01 – 0.78) | -0.20 (-1.12 – 0.71) | -0.26 (-1.23 – 0.71) |
| Sex (f) | – | – | 3.03 (-3.99 – 10.05) | 4.28 (-3.05 – 11.62) | 4.58 (-3.00 – 12.17) |
| CMV-positiv | – | – | – | -2.33 (-9.44 – 4.79) | -2.23 (-9.44 – 4.98) |
| ΔVisceral fat (%) | – | – | – | – | 0.21 (-0.86 – 1.27) |
| Adjusted R² | 0.09* | 0.07 | 0.06 | 0.05 | 0.03 |
|  |  |  |  |  |  |
| ΔCCL2 | β (95% CI ) | β (95% CI ) | β (95% CI ) | β (95% CI ) | β (95% CI ) |
| ΔHand grip | -7.67 (-14.99 – -0.35)* | -7.45 (-14.94 – -0.02)* | -6.60 (-14.20 – 1.00) | -7.81 (-15.66 – -0.02)* | -7.79 (-15.75 – 0.16) |
| Age (baseline) | – | 1.51 (-6.28 – 9.30) | 1.20 (-6.58 – 9.00) | 1.61 (-6.31 – 9.52) | 1.46 (-7.00 – 9.87) |
| Sex (f) | – | – | 35.33 (-25.62 – 96.28) | 25.70 (-37.87 – 89.28) | 26.51 (-39.33 – 92.35) |
| CMV-positiv | – | – | – | 39.82 (-21.08 – 101.45) | 40.08 (-22.52 – 102.67) |
| ΔVisceral fat (%) | – | – | – | – | 0.55 (-8.71 – 9.81) |
| Adjusted R² | 0.07* | 0.05 | 0.06 | 0.08 | 0.05 |

Table S6. Hierarchical linear regression models assessing the association between changes in Δ Grip strength and changes in T-cell subset distributions and circulating inflammatory and regulatory cytokines parameters.

**Note:** Standardized regression coefficients (β) with 95% confidence intervals (CI) are shown. Δ indicates change over the 3-year period (follow-up minus baseline). Adjusted R² represents variance explained by the model. * indicates p < 0.05, ** p < 0.01, *** p < 0.001. **Abbrevations:** CCL: C-C motif chemokine ligand; CMV: cytomegalovirus serostatus; EMRA: effector memory T cells re-expressing CD45RA.

Table S7. Hierarchical linear regression models assessing the association between changes in Δ physical activity and changes in T-cell subset distributions and circulating regulatory cytokines parameters.

|  | Modell 1 | Modell 2 | Modell 3 | Modell 4 | Modell 5 |
| --- | --- | --- | --- | --- | --- |
| ΔCD4^+^ EMRA | ß (95% CI) | ß (95% CI) | ß (95% CI) | ß (95% CI) | ß (95% CI) |
| ΔMET | -7.16x10^-4^ (-0.001 – -4.62x10^-5^)* | -7.53x10^-4^ (-0.001 – -5.06x10^-5^)* | -7.67x10^-4^ (-0.001 – -5.67x10^-5^)* | -9.21x10^-4^ (-0.002 – -1.83x10^-4^)* | -9.39x10^-4^ (-0.002 – -1.88x10^-4^)* |
| Age (baseline) | – | -0.09 (-0.53– 0.35) | -0.06 (-0.52– 0.40) | -0.07 (-0.53– 0.40) | -0.11 (-0.60– 0.39) |
| Sex (f) | – | – | -1.19 (-6.76 – 2.38) | -1.60 (-5.31 – 2.10) | -1.56 (-5.31 – 2.20) |
| CMV-positiv | – | – | – | -2.31 (-5.78 – 1.16) | -2.32 (-5.83 – 1.19) |
| ΔVisceral fat (%) | – | – | – | – | 0.15 (-0.41 – 0.70) |
| Adjusted R² | 0.09* | 0.07 | 0.06 | 0.09 | 0.07 |
|  |  |  |  |  |  |
| ΔIL-1ra | ß (95% CI) | ß (95% CI) | ß (95% CI) | ß (95% CI) | ß (95% CI) |
| ΔMET | -0.04 (-0.08 – -9.48x10^-4^)* | -0.03 (-0.07 – 0.01) | -0.03 (-0.07 – 0.01) | -0.03 (-0.07 – 0.01) | -0.03 (-0.07 – 0.01) |
| Age (baseline) | – | 20.19 (-3.33 – 43.70) | 22.18 (-1.89 – 46.25) | 23.07 (-2.25 – 48.38) | 24.69 (-2.41 – 51.79) |
| Sex (f) | – | – | -80.87 (-269.51 – 107.77) | -95.82 (-297.88 – 106.19) | -97.67(-302.94 – 107.60) |
| CMV-positiv | – | – | – | -15.24 (-204.51 – 174.03) | -14.76 (-206.86 – 177.34) |
| ΔVisceral fat (%) | – | – | – | – | -5.69 (-35.94 – 24.56) |
| Adjusted R² | 0.08* | 0.13* | 0.13 | 0.10 | 0.08 |

**Note:** Standardized regression coefficients (β) with 95% confidence intervals (CI) are shown. Δ indicates change over the 3-year period (follow-up minus baseline). Adjusted R² represents variance explained by the model. * indicates p < 0.05, ** p < 0.01, *** p < 0.001. **Abbrevations:** CMV: cytomegalovirus serostatus; EMRA: effector memory T cells re-expressing CD45RA; IL-1ra: interleukin-1 receptor antagonist; MET: metabolic equivalent of task.
